# Targeting Tumor Angiogenesis with the Selective VEGFR-3 Inhibitor EVT801 in Combination with Cancer Immunotherapy

**DOI:** 10.1158/2767-9764.CRC-22-0151

**Published:** 2022-11-29

**Authors:** Michael R. Paillasse, Michael Esquerré, Florie A. Bertrand, Céline Poussereau-Pomié, Mélanie Pichery, Virgile Visentin, Geneviève Gueguen-Dorbes, Florence Gaujarengues, Pauline Barron, Gaelle Badet, Anne Briaux, Pierre-Benoit Ancey, David Sibrac, Eric Erdociain, Dennis Özcelik, Jérôme Meneyrol, Valérie Martin, Anne Gomez-Brouchet, Janik Selves, Philippe Rochaix, Maxime Battistella, Céleste Lebbé, Jean-Pierre Delord, Frédérique Dol-Gleizes, Françoise Bono, Isabelle Blanc, Antoine Alam, Ian Hunneyball, Mark Whittaker, Pierre Fons

**Affiliations:** 1 Evotec France, Campus Curie, Toulouse CEDEX, France.; 2 Evotec SE, Manfred Eigen Campus, Hamburg, Germany.; 3 Sanofi, Chilly-Mazarin, France.; 4 Institut Universitaire du Cancer Toulouse Oncopole (IUCT-O), Toulouse, Occitanie, France.; 5 Université de Paris, Department of Pathology, AP-HP Hôpital Saint Louis, INSERM U976, Paris, France.; 6 Université de Paris, Department of Dermatology, AP-HP Hôpital Saint Louis, INSERM U976, Paris, France.; 7 Evotec ID, Lyon, France.; 8 Evotec Ltd, Oxfordshire, United Kingdom.

## Abstract

The receptor tyrosine kinase VEGFR-3 plays a crucial role in cancer-induced angiogenesis and lymphangiogenesis, promoting tumor development and metastasis. Here, we report the novel VEGFR-3 inhibitor EVT801 that presents a more selective and less toxic profile than two major inhibitors of VEGFRs (i.e., sorafenib and pazopanib). As monotherapy, EVT801 showed a potent antitumor effect in VEGFR-3–positive tumors, and in tumors with VEGFR-3–positive microenvironments. EVT801 suppressed VEGF-C–induced human endothelial cell proliferation *in vitro* and tumor (lymph)angiogenesis in different tumor mouse models. In addition to reduced tumor growth, EVT801 decreased tumor hypoxia, favored sustained tumor blood vessel homogenization (i.e., leaving fewer and overall larger vessels), and reduced important immunosuppressive cytokines (CCL4, CCL5) and myeloid-derived suppressor cells (MDSC) in circulation. Furthermore, in carcinoma mouse models, the combination of EVT801 with immune checkpoint therapy (ICT) yielded superior outcomes to either single treatment. Moreover, tumor growth inhibition was inversely correlated with levels of CCL4, CCL5, and MDSCs after treatment with EVT801, either alone or combined with ICT. Taken together, EVT801 represents a promising anti(lymph)angiogenic drug for improving ICT response rates in patients with VEGFR-3 positive tumors.

**Significance:**

The VEGFR-3 inhibitor EVT801 demonstrates superior selectivity and toxicity profile than other VEGFR-3 tyrosine kinase inhibitors. EVT801 showed potent antitumor effects in VEGFR-3–positive tumors, and tumors with VEGFR-3–positive microenvironments through blood vessel homogenization, and reduction of tumor hypoxia and limited immunosuppression. EVT801 increases immune checkpoint inhibitors’ antitumor effects.

## Introduction

Angiogenesis and lymphangiogenesis are dynamic processes during embryogenesis, but they are largely absent under normal physiologic postnatal conditions (1, 2). In the adult, angiogenesis and lymphangiogenesis occur only during certain pathologic conditions such as inflammation, tissue repair, and tumorigenesis (3). Cancer cells induce angiogenesis and lymphangiogenesis to establish a specific stromal tumor microenvironment (TME). One of the hallmarks of the TME is hypoxia, which is considered to be a poor prognostic marker for several cancer types (4). Targeting the hypoxic TME, and the cancer redox landscape in general, is a growing area of interest for the development of novel pharmaceuticals in cancer therapy (5). Notably, hypoxia is associated with development of resistance toward current therapies, including immune checkpoint therapies (ICT; refs. 6, 7). Hypoxia creates conditions in the TME that are not suitable for immune cells, thus interfering with immune cell signaling, and eventually leading to immune tolerance and immune escape of the tumor (8). In line with this, previous research highlighted the interlocked nature between lymphangiogenesis, TME, and tumor immunity by demonstrating that tumor lymphangiogenesis is associated with immunosuppression or immune tolerance (9). Besides promoting tumor heterogeneity, plasticity, and immunosuppression, the hypoxic TME plays also a crucial role in metastatic progression (10). In addition, a growing body of evidence demonstrated that tumor-induced lymphangiogenesis is a predictive indicator of metastasis to lymph nodes (11). Moreover, research and clinical evidence demonstrated that the occurrence of primary tumors and, in particular, metastases is associated with high mortality rates in patients with cancer (12).

VEGFRs (VEGFR-1, -2, and -3) are part of the large group of receptor tyrosine kinases (RTK; ref. 13). VEGFR-1 and VEGFR-2, when bound to VEGF-A, are mainly involved in angiogenesis, whereas VEGFR-3, when bound to VEGF-C and VEGF-D, plays a major role in lymphatic vessel development (14). Binding of VEGF-C or VEGF-D induces dimerization and transphosphorylation of VEGFR-3, which activates the RAS-RAF-1-MEK-ERK cascade, promoting lymphangiogenesis. The crucial involvement of VEGFRs and their associated signaling pathways in the orchestration of angiogenesis and lymphangiogenesis renders VEGFRs an attractive target for the treatment of tumors and the prevention of metastasis (15, 16).

As of today, 11 multi-RTK inhibitors with antiangiogenic properties are used for the treatment of advanced cancers (17), notably sorafenib and sunitinib that inhibit a large number of kinases (e.g., FGF/FGFR, cKIT, cMET, RET, PDGF/PDGFR-α/-β, FLT3) as well as VEGFR-1 and -2, and to a lesser extend VEGFR-3 (18). However, therapeutic doses and regimen are limited by the substantial level of adverse effects, for example, hypertension, proteinuria, hand-foot syndrome, anorexia, and fatigue (19). Many of those adverse events were also reported from the use of the monoclonal anti-VEGF-A antibody bevacizumab, which inhibits VEGFR-2–mediated angiogenesis as well (20). Although antiangiogenic RTK inhibitors are usually associated with good response rates and short-term efficacy, their impact on overall survival is limited (21). This can be explained, at least in part, by the limited doses and intermittent regimen that only yield partial or transient inhibition of VEGFR-3, and thus permit lymphangiogenesis as escape mechanism for the tumor (22). Hence, drug development efforts that aim to obtain selective VEGFR-3 inhibitors may help to overcome these limitations. For instance, the pharmaceutical company Eli Lilly developed IMC-3C5, a monoclonal anti-VEGFR-3 antibody, which showed a good safety profile in the phase Ia and/or Ib clinical trial without adverse effects such as hypertension or hand-foot syndrome, but eventually demonstrated only limited efficacy in patients with nonselected colon cancer (23, 24). Notably, EVT801 and IMC-3C5 differ in their target profile: Each are capable of inhibiting VEGFR-3 homodimers but only EVT801 can inhibit VEGFR-3:VEGFR-2 heterodimers (25).

We and others showed previously that selective inhibition of the VEGFR-3 pathway with the RTK inhibitor SAR131675 affected the TME, reduced lymphangiogenesis, decreased immunosuppressive cell populations, and thus led to reduction of tumor growth and metastasis (26–28). Despite the promising findings, SAR131675 development was terminated during preclinical development due to adverse metabolic effects. Substantial efforts in medicinal chemistry were undertaken to identify a compound that shows the same efficacy and selectivity as SAR131675 but without the aforementioned adverse effects. Eventually, these efforts yielded EVT801, whose selectivity, *in vitro* and *in vivo* activities, alone and combined with immune checkpoint inhibitors are presented herein.

## Materials and Methods

### Reagents and Proteins

EVT801 and its main metabolite were prepared within Evotec. Sorafenib (QA-5261) and pazopanib (QA-8519) were purchased from Combi-Blocks. N-diethylnitrosamine (73861) was obtained from Sigma-Aldrich. VEGF-C (R 20-015) was purchased from Reliatech and VEGF-D (622-VD) from R&D Systems. The VEGFR-1 tyrosine kinase domain was obtained from Upstake, VEGFR-2 was prepared in Sanofi, and VEGFR-3 was purchased from Cell Signaling Technology.

### Cell Lines

Human tumor cell lines were obtained from the German Collection of Microorganisms and Cell Cultures GmbH (DSMZ) or the ATCC, and cultured without further authentication in a humidified incubator at 37°C and 5% CO_2_. Cell viability was determined by trypan blue exclusion and always exceeded 95%. HEK293T cells (ATCC CRC-11268) were maintained in MEM (Invitrogen) supplemented with FCS (Gibco Laboratories), and 2 mmol/L Glutamax (Gibco Laboratories). The adult human lung squamous cell carcinoma cell line NCI-H1703 (ATCC CRL-5889) was cultured in RPMI1640 (Gibco Laboratories) supplemented with 10% heat-inactivated FCS, 100 IU/mL penicillin (Gibco Laboratories) and 100 mg/mL streptomycin (Gibco Laboratories). Adult human lymphatic microvascular endothelial cells (hLMVEC; CC2810, Cambrex) were maintained as described previously (29). Cells were tested and authenticated in the laboratory for the expression of VEGFR-3. The murine cell line BNL 1ME A.7R.1 (ATCC TIB75) was derived from the epithelial cell line BNL CL.2 (ATCC TIB73), which originated from a normal BALB/c mouse embryonic liver, by transformation with methylcholanthrene epoxide. BNL cells were transduced with rLV.EF1.mFLT4 lentiviral particles (Vectalys). Cells were cultivated in DMEM (Invitrogen), supplemented with 10% heat-inactivated FCS and 2 mmol/L Glutamax. The murine cell line CT26 T2 is derived from the N-nitroso-N-methylurethane–induced, undifferentiated colon carcinoma cell line CT26.WT (ATCC CRL-2638). Cells were maintained in RPMI1640 containing 10% FCS, 2 mmol/L Glutamax, 1 x non-essential amino acid (Invitrogen), 1 x natrium pyruvate (Invitrogen), 100 IU/mL penicillin, and 100 mg/mL streptomycin. 4T1 mouse mammary adenocarcinoma cells (ATCC CRL-2539) were maintained in RPMI1640 containing 10% heat-inactivated FCS and 2 mmol/L Glutamax as described previously (26).

### Human Tissues

Human tissues were obtained from Institut Universitaire du Cancer Toulouse Oncopole (IUCT-O) and Hôpital Saint-Louis.

### Histology

After fixation with 10% formalin solution for 24 hours, tumors and lungs were processed using a tissue processor (ASP300, Leica). The dehydration step was carried out in ethanol baths with stepwise increasing concentration of ethanol (i.e., 70%, 80%, 95%, 95%, 100%, 100%) for 1 hour per step. The samples were then immersed in three xylene baths for each 1 hour, followed by three paraffin baths at 58°C for 1.5 hours. Then, each tissue sample was embedded in paraffin blocks with a paraffin embedding module (EG1160, Leica). Representative microsections (thickness of 5 μm) of the zone of interest were processed for IHC, hematoxylin-eosin staining, or ISH and imaging (see below).

### IHC

IHC stainings with anti-mouse VEGFR-3, anti-pimonidazole (hypoxyprobe), anti-mouse CD8α, and anti-mouse CD31 were performed on a VENTANA discovery XT automated staining instruments (Ventana Medical Systems). Anti-human VEGFR-3 and anti-human carbonic anhydrase IX (CAIX) stainings were performed on a VENTANA discovery Ultra automated staining instruments (Ventana Medical Systems) with VENTANA reagents as per manufacturer's protocol. Paraffin was removed from slides using Discovery wash solution (Roche, 07311079001) for three cycles of 8 minutes at 69°C. Epitope retrieval was accomplished with discovery CC1 solution (ACDBio, 06414575001) or UltraCC2 solution (ACDBio, 05424542001) at high temperature (i.e., 95°C to 100°C) for 32 to 40 minutes. Slides were then developed using the VENTANA multimer UltraMap anti-rabbit HRP (Roche, 05269717001) for mouse VEGFR-3, mouse CD8α, mouse CD31, and human CAIX IHC according to the manufacturer's instructions. Slides were then developed using the VENTANA multimer OmniMap anti-Rabbit HRP (Roche, 05269679001) for human VEGFR-3, and pimonidazole IHC according to the manufacturer's instructions. Slides were then counterstained with hematoxylin II (Roche, 05277965001) for 8 minutes, followed by Bluing reagent (Roche, 05266769001) for 4 minutes. Images (2 per tumor and per staining were obtained unless specified otherwise) were acquired using NanoZoomer 2.0 slide scanner (Hamamatsu) using bright field.

### Hematoxylin-eosin Staining

Slides were deparaffinized in xylene bath, rehydrated in several baths of 100% ethanol and water, and stained with Mayer hematoxylin (Diapath, C0303) for 5 minutes. After a tap water wash and a second wash with distilled water, the slides were stained with aqueous eosin (Diapath, C0363), adjusted with glacial acetic acid to a pH between 4.5 and 5.5. After rinsing and dehydration with 70% ethanol, slides were mounted. All these steps were performed in a staining device (Tissue-Tek Prisma, Sakura).

### ISH and Imaging

ISH was performed on a VENTANA discovery Ultra automated staining instruments (Ventana Medical Systems), using VENTANA reagents according to the manufacturer's instructions. Paraffin was removed from slides using Discovery wash solution (Roche, 07311079001) for three cycles of 8 minutes at 69°C. Epitope retrieval was accomplished with discovery CC1 solution (Roche, 06414575001) or at 97°C for 24 minutes. Enzymatic digestion of proteins, hybridization and amplification of *FLT4*-Tv1 probe (ACDBio, 481061), negative control probe DapB (ACDBio, 312039) and positive control probe Hs-PPIB (ACD Bio, 313909) were performed with RNAscope 2.5 versus Reagent Kit-BROWN (ACDBio, 322200) according to manufacturer's instructions. Slides were then counterstained with hematoxylin II (Roche, 05277965001) for 8 minutes, followed by Bluing reagent (Roche, 05266769001) for 4 minutes. Images were acquired using Nanozoomzer 2.0 slide scanner (Hamamatsu) using bright field.

### Animal Studies

All animal treatment procedures described in this study were approved by the Animal Care and Use Committee of Sanofi or Evotec France Ethical Committee. The facility is accredited by the French Ministry of Agriculture and by the Association for Assessment and Accreditation of Laboratory Animal Care International. Female BALB/cByJRj mice and male C3/H mice were obtained from Janvier labs, Le Genest St Isle, France. RIP1.Tag2 mice with a C57Bl/6J background were obtained from Charles River Laboratories, Les Oncins, France. NOD.CB17 Prkdc^scid^/NCrHsd homozygous mice and outbred athymic (nu/nu) female HSD mice were obtained from Envigo.

### RT-001-HAM Subcutaneous Patient-derived xenograft (PDx) Tumor Mouse Model

Patient-derived tumors of the same passage were transplanted subcutaneously outbred athymic (nu/nu) female HSD mice. When tumor volume reached 726 to 1,437 mm^3^, 4 donor mice were sacrificed by cervical dislocation, and tumors were aseptically excised and dissected. After removing necrotic areas, tumor fragments of approximately 20 mm^3^ were transferred to culture medium before grafting. A total of 108 mice were anesthetized with 100 mg/kg ketamine hydrochloride (Virbac) and 10 mg/kg xylazine (Bayer). Then, a tumor fragment was placed in the subcutaneous tissue of an incision at the level of the interscapular region as described previously (30). All mice from the same experiment were implanted on the same day. A total of 54 mice with RT-001-HAM established growing tumor (P6.0.0/0) between 62.5 and 196 mm^3^ were allocated according to tumor volume, ensuring homogenous median and mean tumor volume in each treatment arm. Treatments with 5 mL/kg vehicle, 30 mg/kg EVT801, or 30 mg/kg pazopanib started 15 days after tumor implantation and continued twice per day until termination 7 days later. Tumor volumes were measured via a caliper three times a week during the treatment period. All animals were weighed at the same time as tumor size measurement. Plasma and fresh tumor samples were collected at different timepoints after final dose at day 7 from all mice.

### 4T1 Mammary Carcinoma Mouse Model

The 4T1 mammary carcinoma mouse models were used as reported previously (26). Briefly, 1 × 10^5^ 4T1 cells were implanted into mammary fat pads of BALB/c mice. Twice daily treatments with 30 mg/kg EVT801 by oral gavage and either 10 mg/kg anti-PD-1 (BioXcell, BP0146) or anti-CTLA-4 (BioXcell, BE0131) weekly commenced when tumors reached 50 mm^3^ and lasted 3 weeks. Tumors were measured two to three times weekly with calipers. The tumor volume (*V*) was calculated using the formula *V* = 0.52 × *a*^2^ × *b* (*a*: smallest tumor diameter; *b*: largest tumor diameter). The tumors, lungs, and axillary lymph nodes were removed at day 21 for treatment with anti-PD-1, and day 28 for treatment with anti-CTLA-4. The tumors and the lungs were embedded in paraffin for histology studies. Intraperitoneal injection (10 mg/kg of antibody diluted at 1 mg/mL) was performed at day 6, day 11, and day 16 for treatment with anti-PD-1, and at day 6, day 11, day 16, and day 21 for treatment with anti-CTL-4 by using a 26G needle fitted to a plastic syringe in the right lower quarter of mice abdomen. The metastatic scoring was determined as follows: (0) no metastasis; (1) 1 to 20 metastases < 50 μm; (2) between 21 and 50 metastases < 50 μm; (3) 1 or more metastases > 50 μmol/L; (4) 1 or more metastases > 200 μmol/L.

### N-diethylnitrosamine–Induced Hepatocarcinoma Mouse Model

After weighing, 38 male C3/H mice received a 10 mg/kg dose of N-diethylnitrosamine (DEN) intraperitoneally, whereas a group of 5 animals was kept without DEN injection as control group. After 12 months, mice that received DEN injection were randomized by weight in two groups of each 19 animals. The mice were dosed daily orally for 2 months either with EVT801 or vehicle. On the day of termination, mice treated with EVT801 received a last dose of 30 mg/kg to take blood and tumor samples for compound concentration determination. After incision of the peritoneal cavity, blood was taken from the vena cava. Following excision and weighing of the liver, the number of tumors per liver was counted. The tumor burden was calculated as the sum of individual tumor volumes for each mouse. No blood sampling was performed on control and vehicle groups. Plasma and tumor samples were stored at −20°C until analysis. Concentration of EVT801 was determined by LC/MS-MS.

### NCI-H1703 Subcutaneous Xenograft Tumor Mouse Model

A total number of 40 homozygous NOD.CB17 Prkdc^scid^/NCrHsd mice were used for the xenograft tumor model. On the day of implantation, NCI-H1703 cells were harvested and suspended at a concentration of 1 × 10^8^ cells per mL in an equal mix of Cultrex:RPMI without supplements. A volume of 100 μL was injected into the right hind flank of each animal. Tumor volumes were monitored until mean tumor volume reached 150 mm^3^. Then, mice were stratified into four treatment groups of each 10 mice and orally dosed twice per day with either EVT801 30 mg/kg and EVT801 vehicle [Soluplus (BASF)/water/hydroxypropylcellulose SL (Nisso America)], or 30 mk/kg pazopanib and pazopanib vehicle (0.5% Hydroxypropyl methylcellulose trimellitate (HPMCT) + 0.1% Tween-80). A period of 8 hours was observed between the first and second daily dose.

### Rip1-Tag2/transgenic Mouse Models

The Rip1-Tag2/transgenic mouse models were used as reported previously (29). Briefly, treatment of mice started at the age of 12 weeks. Mice were treated daily for 16 days, and volume of each tumor was measured and calculated as described for the 4T1 mammary carcinoma mouse model below. The tumor burden was calculated as the sum of individual tumor volumes for each mouse. For the survival study, daily treatment started at the age of 12 weeks, and mice were monitored daily to detect moribund mice for euthanasia. CD31-positive vessel density within the tumors were quantified by density index (1 vessel/mm^2^) measured using the Definiens software. Individual density indices were plotted as the mean ± SD for each group. Statistical significance was assessed by Kruskall–Wallis followed by Dunnet multiple comparison test.

### CT26 Ectopic Tumor Mouse Model

BALB/c mice were anesthetized with ketamine (100 mg/kg) combined with xylazine (10 mg/kg) via intraperitoneal injection. A total of 5 × 10^4^ CT26 tumor cells were suspended in 200 μL of Matrigel matrix, and then inoculated in the flank of legs. After implantation, mice were randomly separated into two groups. One group received EVT801 by oral gavage and anti-PD-1 by intraperitoneal injection in a volume of 10 mL/kg, whereas the mice in the second group served as control, and were injected with IgG isotype. PD-1 was injected at day 11, day 14, and day 18 after CT26 inoculation. EVT801 was administrated daily by oral route at 30 mg/kg from day 11 until day 21. Tumor volume was measured on days 11, 13, 15, 18, 19 and 21 following tumor cell injection. The selected groups received vehicle or EVT801 orally in a volume of 10 mL/kg. At day 21, 60 mg/kg hypoxyprobe was injected 30 minutes before sacrifice of mice. The tumors were embedded in paraffin for histology studies.

### Immune Profiling via Flow Cytometry

Whole blood samples were treated twice with ACK lysing buffer (Thermo Fisher Scientific, A1049201) to lyse red blood cells. Cell suspension were incubated for 15 minutes on ice in blocking buffer (FACS buffer: PBS 1 X, 1% FBS, 5 mmol/L Ethylene Diamine Tetra acetic Acid (EDTA) containing 3% rat serum, 3% mouse serum, 1:660 FC blocking antibody). Cell suspension were stained with an antibody mix either for myeloid-derived suppressor cell (MDSC) staining [anti-CD45 PE-Vio770 (Miltenyi Biotec, 130-110-799), anti-CD11b APC (eBioscience, 17-0112-82), anti-Ly6C AF700 (BD, 561237), anti-Ly6G BV421 (BD, 562737), anti-F4/80 BV510 (BD, 743280), anti-IA/I-E (MHCII), PercpCy5.5 (BD, 562363), anti-CD124 PE (BioLegend, 144804) or anti-rat IgG2bPE (Ozyme, BLE400608) isotype control, and the green fluorescent reactive dye (Thermo Fisher Scientific, L34960)], or for T-cell staining [anti-CD45 PE-Vio770 (Miltenyi Biotec, 130-110-799), anti-Thy1 APC-Cy7 (BioLegend, 105328), anti-CD4 PercpVio-700 (Miltenyi Biotec, 130-109-497), anti-CD8 BV510 (BD, 563068), anti-CD69 BV421 (BD 562920) or anti-rat IgG1a-BV421 (BD, 562919), and the green fluorescent reactive dye (Thermo Fisher Scientific, L34960)]. After incubation for 30 minutes at 4°C in the dark, cells were washed and suspended in FACS buffer. A total of 50 μL of CountBright Absolute counting beads (Thermo Fisher Scientific, C36950) were added just before cell acquisition on a FACS Fortessa X20 (Becton Dickinson). Analysis was performed with the FlowJo software. For calculation of cell concentration, the following formula was used: Concentration of cells/μL = (number of cell events/number of bead events) × (assigned bead count of the lot/volume of analyzed sample). Monocytic MDSCs (M-MDSC) were characterized as MHC-II^neg^/CD11b^+^/Ly6C^+^/Ly6G^neg^, whereas polymorphonuclear MDSCs (PMN-MDSC) were characterized as MHC-II^neg^/CD11b^+^/Ly6C^neg^/Ly6G^+^, as described previously (31). Among those populations, CD124^+^ cells were considered as the activated subpopulations (32).

### Plasmatic Factor Dosage by MesoScale Discovery

Blood was centrifuged at 8,000 rpm, for 5 minutes, at 4°C and the plasma was collected by harvesting the supernatant and stored at −80°C. Plasma samples were thawed at 4°C extemporaneously for MesoScale Discovery (MSD) analysis on a U-PLEX-4 Assay coated plate (U-PLEX Mouse KC, U-PLEX Mouse MIP-1α/CCL3, U-PLEX Mouse MIP-1β/CCL4, U-PLEX Mouse Rantes/CCL5 K15069L-1, Meso Scale Discovery). Analysis was performed following manufacturer's instructions.

### Illustration Tools

Graphs were created with Prism GraphPad 8.3.1, and figures were created with Inkscape 1.02-2.

### Statistical Analysis and Experimental Design

Statistical analyses were performed using GraphPad Prism 8.3.1 software, and a *P* value of < 0.05 was deemed as statistical significant. Data are presented as mean with SD, unless otherwise specified. Statistical comparisons of two groups were performed using nonparametric unpaired Student *t* test for two-tailed *P* value unless otherwise specified (ns, *P* ≥ 0.05; *, *P* < 0.05; **, *P* < 0.01; ***, *P* < 0.001; ****, *P* < 0.0001). For statistical comparisons with more than two groups, an ordinary one-way ANOVA statistical test followed by Dunnett multiple comparison test was performed, and a two-way ANOVA followed by Tukey multiple comparison test in case of repeated measures. For each timepoint, the mean tumor volume in the treated groups is compared with the vehicle-treated group. Differences between compound and vehicle-treated mice were considered significant with *P* values as listed above. For statistical analysis in the survival study, the log-rank test method was used for the comparison of the EVT801-treated group to the vehicle-treated group. Unpaired *t* tests (Welch *t* tests) were performed for FACS and MSD analysis.

Unless specified otherwise, all biochemical and cellular experiments were performed three times, and data from the median experiment are presented. Unless specified otherwise, *in vivo* experiments, including pharmacokinetics and pharmacodynamics, were performed twice, and the presented data are the same as selected for the preparation of the Clinical Trial Application (NCT05114668).

## Results

### EVT801 is a Novel Selective VEGFR-3 Inhibitor, Capable of Modulating Lymphangiogenesis and Angiogenesis

**FIGURE 1:**
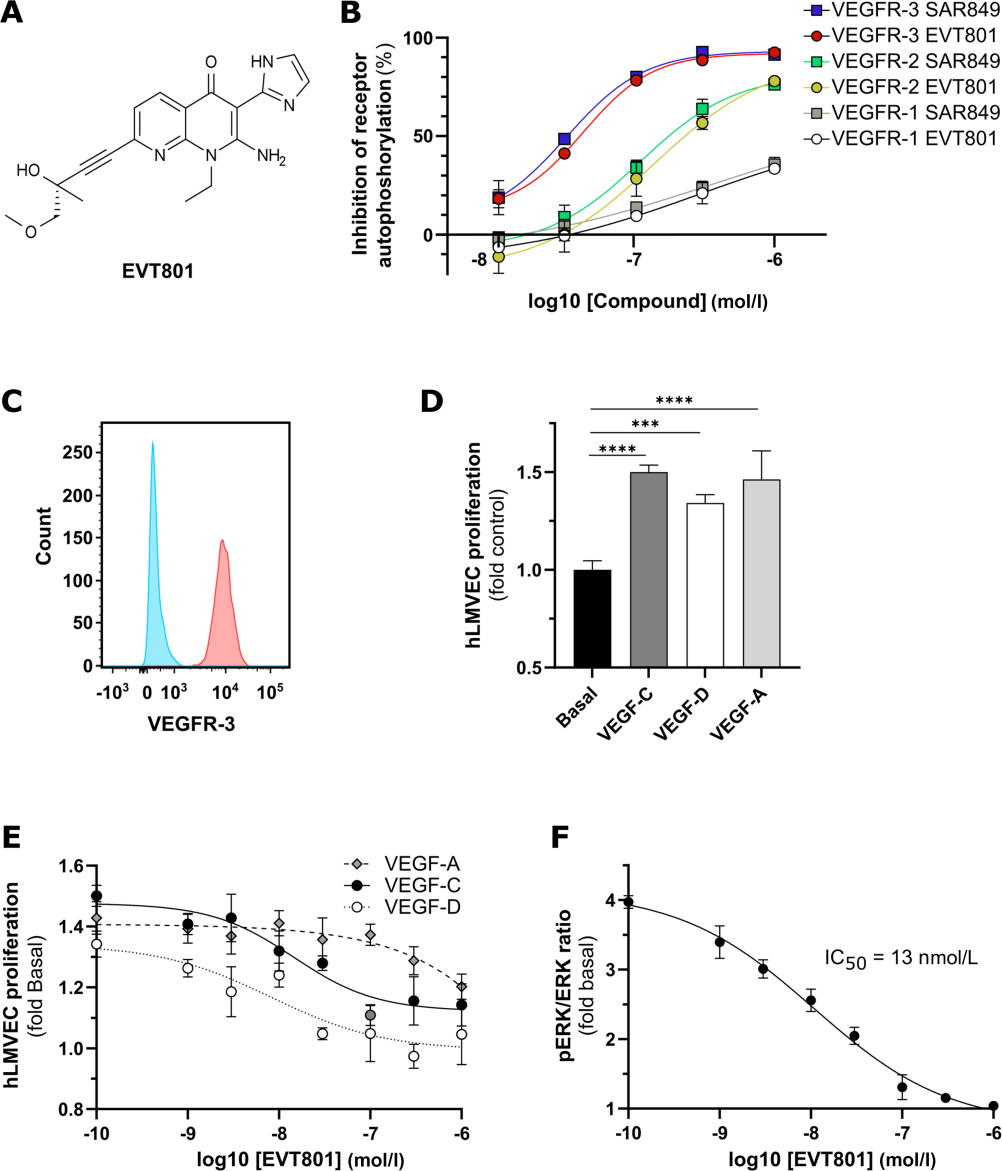
EVT801 is a novel selective VEGFR-3 inhibitor preventing (lymph)angiogenesis. **A,** Chemical structure of EVT801. **B,** Dose–response curve of receptor inhibition by EVT801 in HEK293 cells, expressing VEGFR-1, VEGFR-2, or VEGFR-3. **C,** Histogram of VEGFR-3 expression in hLMVEC. **D,** Stimulation of hLMVEC proliferation by VEGF-A, VEGF-C, and VEGF-D. **E,** Dose–response curve of the inhibition of VEGF-A-, VEGF-C-, and VEGF-D-induced hLMVEC proliferation by EVT801. **F,** Dose–response curve of the inhibition of VEGF-C–induced ERK1/2 phosphorylation by EVT801 in hLMVEC.

Given the pivotal role of VEGFR-3 in cancer development, we initiated a lead-optimization program to identify a selective small-molecule inhibitor of this particular RTK. These efforts led to a promising compound, termed EVT801 (Fig. 1A). We assessed its potency to inhibit VEGFR-3 autophosphorylation, and its selectivity over VEGFR-1 and VEGFR-2 in biochemical and cellular assays (i.e., HEK293 cells transiently expressing the appropriate receptor). EVT801 showed an inhibitory activity in the low nanomolar range and was selective in both assays (IC_50_ in biochemical assays: 11 nmol/L for VEGFR-3, 396 nmol/L for VEGFR-1, 130 nmol/L for VEGFR-2; IC_50_ in cellular assays: 39 nmol/L for VEGFR-3, 2130 nmol/L for VEGFR-1, 260 nmol/L for VEGFR-2). In addition, the inhibitory effect of EVT801 on VEGFR-3 autophosphorylation was assessed in various mammalian species, with an IC_50_ in cellular assays ranging from 151 nmol/L for the mouse receptor to 32 nmol/L for the monkey protein (Supplementary Table S1). Further studies also confirmed the high selectivity of EVT801 across kinases, various receptors and ion channels. Subsequent metabolic studies indicated that EVT801 undergoes O-demethylation in the cell, giving rise to the active metabolite SAR401849, which is as potent and selective as EVT801 (Supplementary Fig. S1A; Supplementary Table S2). EVT801 and the active metabolite showed almost identical dose–response curves and exhibited substantial selectivity for VEGFR-3 over VEGFR-1 (with maximum inhibition below 50%) and VEGFR-2 in the HEK293T cellular autophosphorylation assay (Fig. 1B). This was also confirmed in Ba/F3 cell lines that were modified to depend on either VEGFR-2 or VEGFR-3 for viability. SAR401849 inhibited VEGFR-2–dependent viability with an IC_50_ of 398 nmol/L, and VEGFR-3–dependent viability with an IC_50_ of 32 nmol/L, EVT801 inhibited viability to a similar degree, that is, IC_50_ of 248 nmol/L for VEGFR-2 and 24 nmol/L for VEGFR-3 (Supplementary Fig. S1B and S1C).

To further evaluate the capacity of EVT801 to prevent proliferation of VEGFR-3–positive cells, we used hLMVECs. First, we validated expression of VEGFR-3 in those cells by flow cytometry, and confirmed expression in at least 98% of live cells (Fig. 1C). Next, we determined that the VEGFR-3 ligands VEGF-C and VEGF-D increased hLMVEC proliferation by 50% and 34%, respectively, and similarly, the VEGFR-2 ligand VEGF-A (VEGF_165_) induced an increase in proliferation by 46% (Fig. 1D). EVT801 was able to inhibit the induction of hLMVEC proliferation in a dose-dependent manner with an IC_50_ of 15 nmol/L for VEGF-C, 8 nmol/L for VEGF-D, and 155 nmol/L for VEGF-A, and a maximum inhibition of 74%, 100%, and 65%, respectively (Fig. 1E). Such inhibitory activity on lymphatic cell proliferation was also observed on blood vessels in a mouse aortic ring assay that was adapted to recapitulate neoangiogenesis (Supplementary Fig. S2). Because binding of VEGF-C to VEGFR-3 results in downstream activation of ERK (33), we sought to assess the phosphorylation state of ERK1 and ERK2 upon treatment with EVT801 as a potential biomarker of activity. Our analysis indicated that EVT801 strongly inhibited VEGF-C–induced ERK1/2 phosphorylation with an IC_50_ of 13 nmol/L (Fig. 1F), which correlated with the observed antiproliferative effect.

### VEGFR-3 is Expressed in Blood Vessels of Kidney Cancer Primary Tumors and Metastases, and in Tumor Cells of Endothelial Malignancies

**FIGURE 2:**
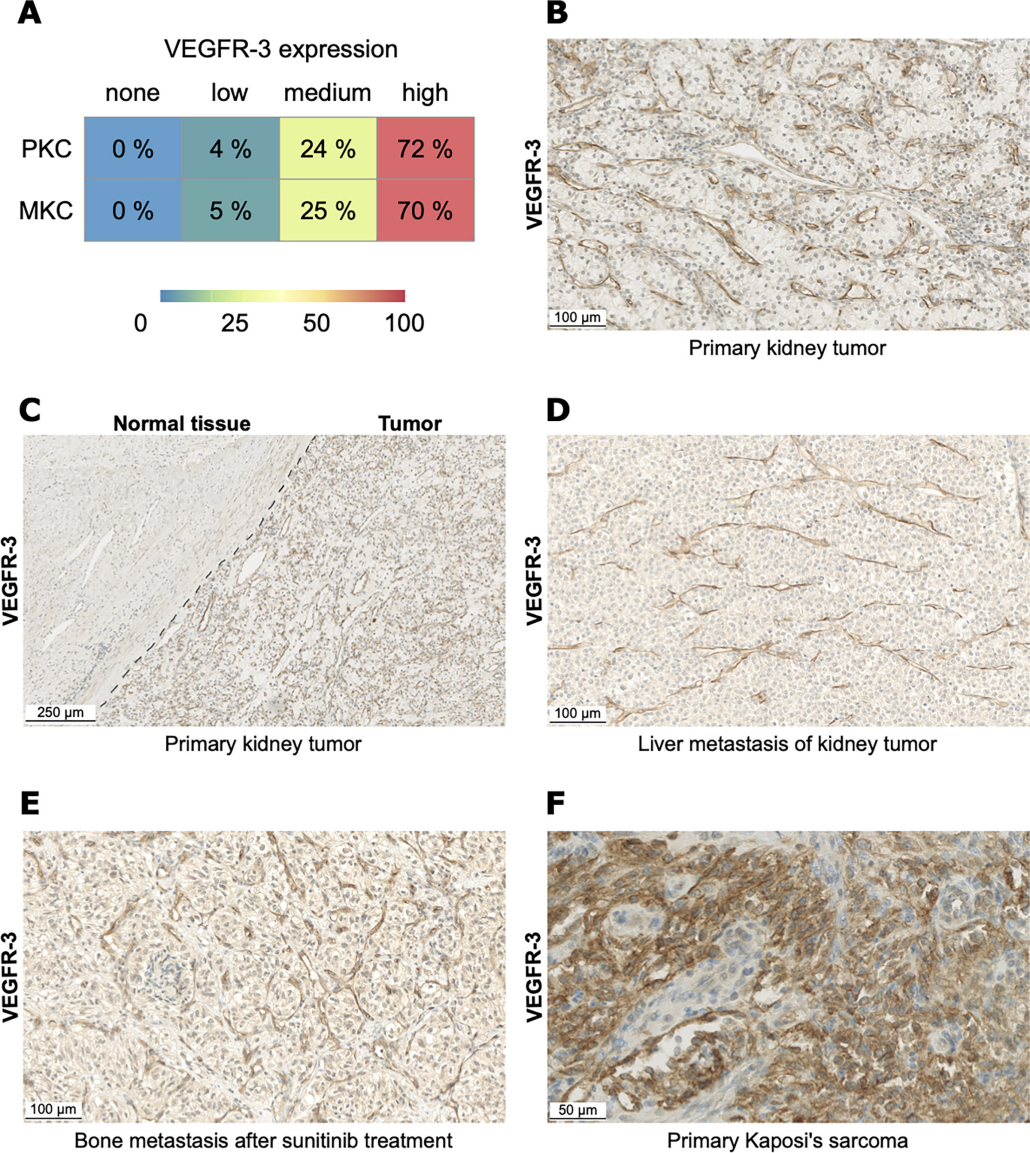
VEGFR-3 expression in kidney cancer cohorts. **A,** Level of VEGFR-3 expression in 29 primary kidney cancer (PKC) samples and 23 metastatic kidney cancer (MKC) samples. **B,** Representative IHC image of VEGFR-3 expression in primary kidney tumor. **C,** Representative IHC image of VEGFR-3 expression in primary kidney tumor and adjacent normal tissue. **D,** Representative IHC image of VEGFR-3 expression in liver metastasis of kidney tumor. **E,** Representative IHC image of VEGFR-3 expression in bone metastasis after treatment with sunitinib. **F,** Representative IHC image of VEGFR-3 expression in primary Kaposi sarcoma.

Literature reports widespread VEGFR-3 expression in several tumors, especially in tumors of the kidney and the liver (34–36). To validate the findings of these reports, we developed a highly specific protocol for VEGFR-3 labeling, readily transferable to clinical centers (see IHC section in the Materials and Methods). We focused our analysis on kidney cancer because multi-RTK inhibitors are part of the standard-of-care therapy for this indication. We assessed VEGFR-3 expression in 29 primary kidney cancer samples and 23 metastatic kidney cancer samples (Fig. 2A). We found that VEGFR-3 expression was predominantly limited to endothelial cells within the kidney tumor (Fig. 2B). Remarkably, we observed a very distinct delineation between the kidney tumor, which showed high VEGFR-3 expression, and the normal adjacent tissue which showed low VEGFR-3 expression (Fig. 2C). Moreover, we used CD34 as broad vessel marker and D2-40 to specifically stain lymphatic vessels, and could see that the tumors contain vessels stained by CD34, most of those coexpress VEGFR-3. In the contrary, tumors are devoid of lymphatic vessels because no D2-40 staining was visible. In the normal adjacent tissue, there were characterized vessels coexpressing CD34 and VEGFR-3 and some coexpressing CD34, VEGFR-3, and D2-40 (Supplementary Fig. S3). Altogether, these findings indicate that VEGFR-3 is expressed in vascular endothelial cells within the tumor as well as in vascular and lymphatic endothelial cells in normal adjacent tissue. In this article, we did not distinguish between the impact of EVT801 on vascular and on lymphatic vessels but instead considered the effect on angiogenesis and lymphangiogenesis globally. In addition, we found that VEGFR-3 expression in metastases from kidney tumors corresponded to the expression pattern of the parent tumor (Fig. 2D). Furthermore, metastases from kidney tumors still showed high expression levels of VEGFR-3 after treatment with the RTK inhibitor sunitinib (Fig. 2E), suggesting that EVT801 administration may also be warranted after failure of other RTK inhibitors. We eventually explored VEGFR-3 expression in cancer types of endothelial origin, such as Kaposi sarcoma, and could confirm that VEGFR-3 was highly expressed in tumor cells and the TME (Fig. 2F) as reported previously (37). This suggests that Kaposi sarcoma and other cancers from endothelial origin are an attractive cancer type for single-agent treatment with EVT801.

### EVT801 Inhibits Proliferation and Growth of VEGFR-3–positive Tumors

**FIGURE 3:**
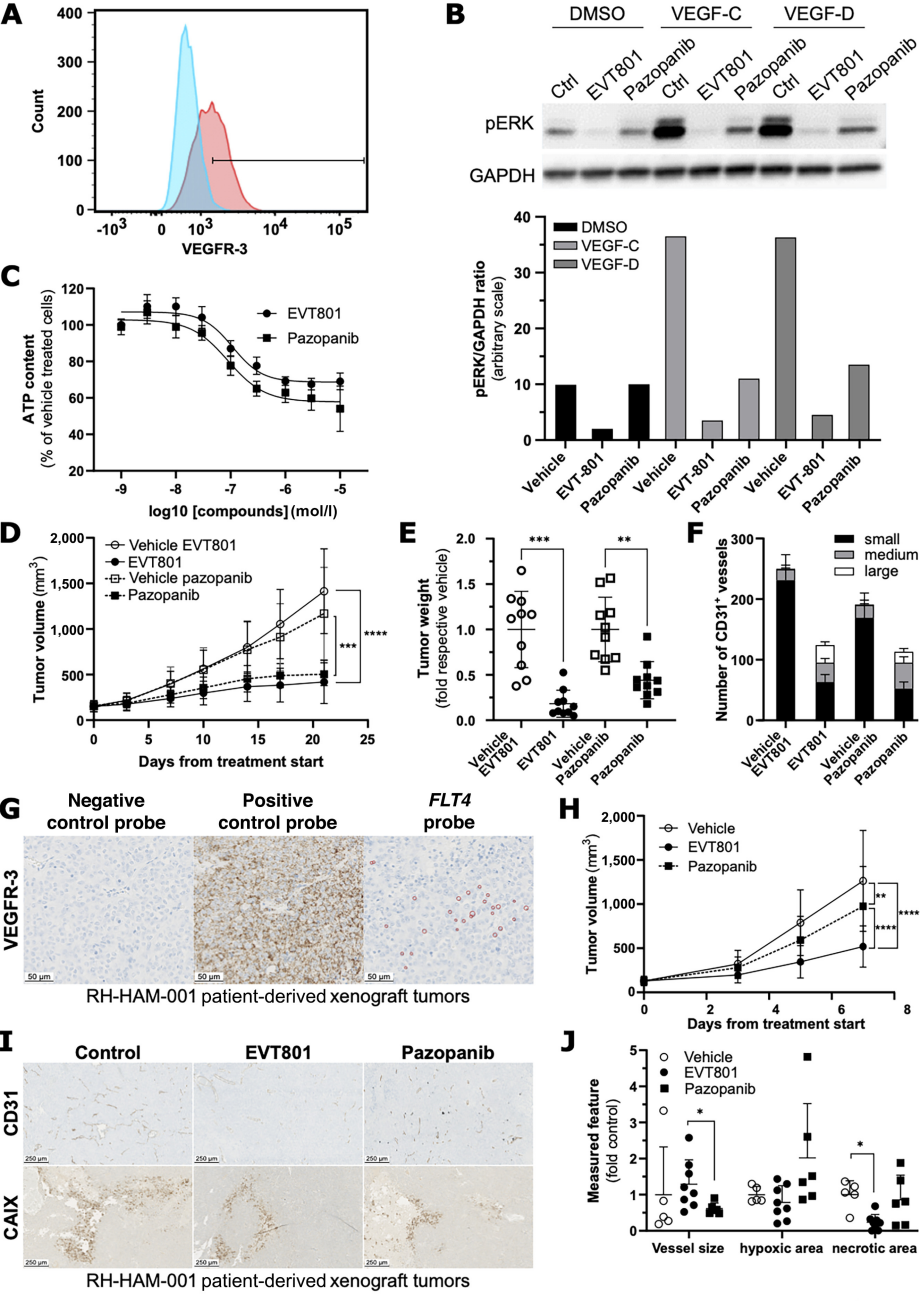
EVT801 inhibits proliferation and tumor growth of VEGFR-3–positive tumors. **A,** Histogram of VEGFR-3 expression in NCI-H1703 cells. **B,** Immunoblot of phosphorylated ERK (pERK) and GAPDH in NCI-H1703 cells in presence of DMSO, VEGF-C, or VEGF-D, and upon treatment with vehicle, EVT801 1 μmol/L or pazopanib 1 μmol/L. Quantitation of pERK/GADPH ratio is depicted in the bar graph below. **C,** Dose–response curves of ATP reduction (as surrogate for NCI-H1703 viability) by EVT801 and pazopanib. **D,** Subcutaneous NCI-H1703 tumor volume progression over time in presence of EVT801 (30 mg/kg twice per day), pazopanib (30 mg/kg twice per day) or vehicles. **E,** Subcutaneous NCI-H1703 tumor weight inhibition at endpoint after treatment with EVT801, pazopanib, or vehicles. **F,** Quantification of CD31-positive blood vessels in NCI-H1703 tumors treated with EVT801, pazopanib, or vehicle. Small vessels have no visible lumen, medium vessels have a surface of <1,000 μm² and large vessels of >1,000 μm². **G,** Representative ISH image of *FLT4* mRNA expression in RH-HAM-001 patient-derived xenograft tumors. Hs-PPIB is used as positive control to validate *FLT4* mRNA staining. **H,** Subcutaneous RH-HAM-001 tumor volume progression over time in presence of EVT801 (30 mg/kg twice per day), pazopanib (30 mg/kg twice per day) or vehicle. **I,** Representative IHC images of blood vessels via CD31 expression (top) and hypoxic zones via CAIX expression (bottom) in RH-HAM-001 tumors. **J,** Quantitation of mean vessel size, hypoxic, and necrotic areas in RH-HAM-001 tumors.

Because no Kaposi cell line was commercially available, we looked for VEGFR-3–positive tumor cell lines using flow cytometry and found that 45% of NCI-H1703 cells expressed VEGFR-3, rendering this cell line a suitable model (Fig. 3A). Next, we interrogated how potently EVT801 interfered with VEGFR-3 signaling in NCI-H1703 cells, and how it compared with the multi-RTK inhibitor pazopanib, which targets mainly VEGFR-1, VEGFR-2, VEGFR-3, FGFR-1, FGFR-3, B-raf, C-kit, and PDGFR, and was approved for the treatment of soft-tissue sarcoma, including Kaposi sarcoma and kidney cancer. EVT801 reduced ERK phosphorylation induced by VEGF-C and VEGF-D better than pazopanib (Fig. 3B), while the two compounds were equally potent to prevent NCI-H1703 viability *in vitro*, with IC_50_ of 109 nmol/L for EVT801 and 90 nmol/L for pazopanib (Fig. 3C). To evaluate the efficacy of EVT801 *in vivo*, we first assessed the relationship between circulating concentrations of EVT801 and ERK phosphorylation and validated that ERK phosphorylation can serve as a pharmacodynamic biomarker for EVT801 treatment. This also validated that a dose regimen of 30 mg/kg twice per day, or regimens producing a similar exposure (AUC; e.g., 100 mg/kg every day, should be used *in vivo* (Supplementary Fig. S4A; Supplementary Table S3). Monitoring of AKT phosphorylation yielded similar results, albeit with higher variability (Supplementary Fig. S4B). Then, we assessed whether these findings could be transferred to subcutaneous NCI-H1703 tumor xenografts. Indeed, oral administration of 30 mg/kg twice per day EVT801 showed a significant inhibition of tumor growth compared with administration of the vehicle [treated/control (T/C) ratio of 24%], as well as did 30 mg/kg twice per day pazopanib compared with its corresponding vehicle group (T/C ratio of 53%; Fig. 3D). An endpoint analysis of the individual tumor weight uncovered that EVT801 decreased tumor weight by 82% compared with vehicle, whereas pazopanib decreased the weight by only 56% compared with vehicle (Fig. 3E). Subsequent IHC imaging of the endothelial cell marker CD31 (38) showed that EVT801 and pazopanib decreased the number of vessels while increasing the proportion of larger ones, which herein is summarized as homogenization (Fig. 3F). In addition, we monitored CAIX, an endogenous marker for hypoxic cells, a hallmark of reduced perfusion in presence of antiangiogenics, and a potential early indicator of tumor escape (39). IHC imaging did not detect CAIX and hence no hypoxic zone neither in the control group nor in any of the treated group (Supplementary Fig. S5).

To validate this in another model of VEGFR-3–positive tumors, we transfected the hepatoma cell line BNL with mouse VEGFR-3 (BNL-R3), and then ectopically injected these BNL-R3 cells into the flanks of mice (Supplementary Fig. S6A). Following injection, we evaluated the outcome of EVT801 treatment at two doses, that is, 10 and 30 mg/kg twice per day. Both doses significantly inhibited tumor growth compared with the vehicle-treated group, with T/C ratio of 31% and 10%, respectively (Supplementary Fig. S6B). EVT801 apparently induced vessel homogenization because 10 and 30 mg/kg treatments decreased number of vessels by 60% and 83%, respectively, whereas the total vessel surface was only decreased by 35% and 29% (Supplementary Fig. S6C and S6D). Furthermore, both doses of EVT801 also decreased hypoxia (Supplementary Fig. S6E) while tumor immunity was altered. Specifically, infiltration of CD3^+^ cells, which are associated with tumor killing (40), was increased in the tumor at the endpoint (Supplementary Fig. S7A). CD8^+^ cells were also enriched in several tumors, but the degree of infiltration did not reach statistical significance (Supplementary Fig. S7B). This finding correlated with decreased circulation of activated CD124^+^ PMN-MDSCs and M-MDSCs, respectively, which are known for their immunosuppressive role (ref. 41; Supplementary Fig. S7C and S7D). In contrast the CD8^+^ T, CD4^+^ T-cell, and respective FoxP3^+^ regulatory T cell subpopulations were not significantly altered (Supplementary Fig. S7E and S7F).

To confirm these findings in a more translational model, we selected a patient-derived xenograft of rhabdomyosarcoma, termed RT-HAM-001 (Xentech SAS). We first determined VEGFR-3 expression using IHC, yielding unspecific (membrane and cytosol) staining in all cells. We then quantified mRNA levels of VEGFR-3 by ISH, and found that 20% of cells in RT-HAM-001 tumors expressed detectable amounts of *FLT4* mRNA (Fig. 3G). We, once again, compared the outcome of EVT801 and pazopanib 30 mg/kg twice per day treatments. Despite the limited VEGFR-3 expression in the tumor cells, we found that EVT801 treatment induced a significant reduction in tumor volume (Fig. 3H). Remarkably, EVT801 performed almost twice as well as pazopanib, according to the respective T/C ratios of 41% and 77%. Subsequent IHC imaging of CD31 showed that both EVT801 and pazopanib marginally decreased the amount of vessels within the tumor; nevertheless, the remaining vessels in the tumor were significantly larger upon EVT801 treatment than upon pazopanib treatment (Fig. 3I and J). In addition, IHC imaging of CAIX showed only limited hypoxic zones, mainly in the surroundings of necrotic areas within the tumor (Fig. 3I). After this short 7-day treatment, differences in hypoxic areas did not achieve statistical significance. Nevertheless, EVT801 appeared to decrease hypoxia in half of the tumors, whereas pazopanib showed opposing effects. This observation aligned with the size of necrotic areas that decreased after EVT801 treatment but remained unaffected after pazopanib treatment (Fig. 3J).

### EVT801 Inhibits Proliferation and Growth of Tumors with VEGFR-3–positive TME

**FIGURE 4:**
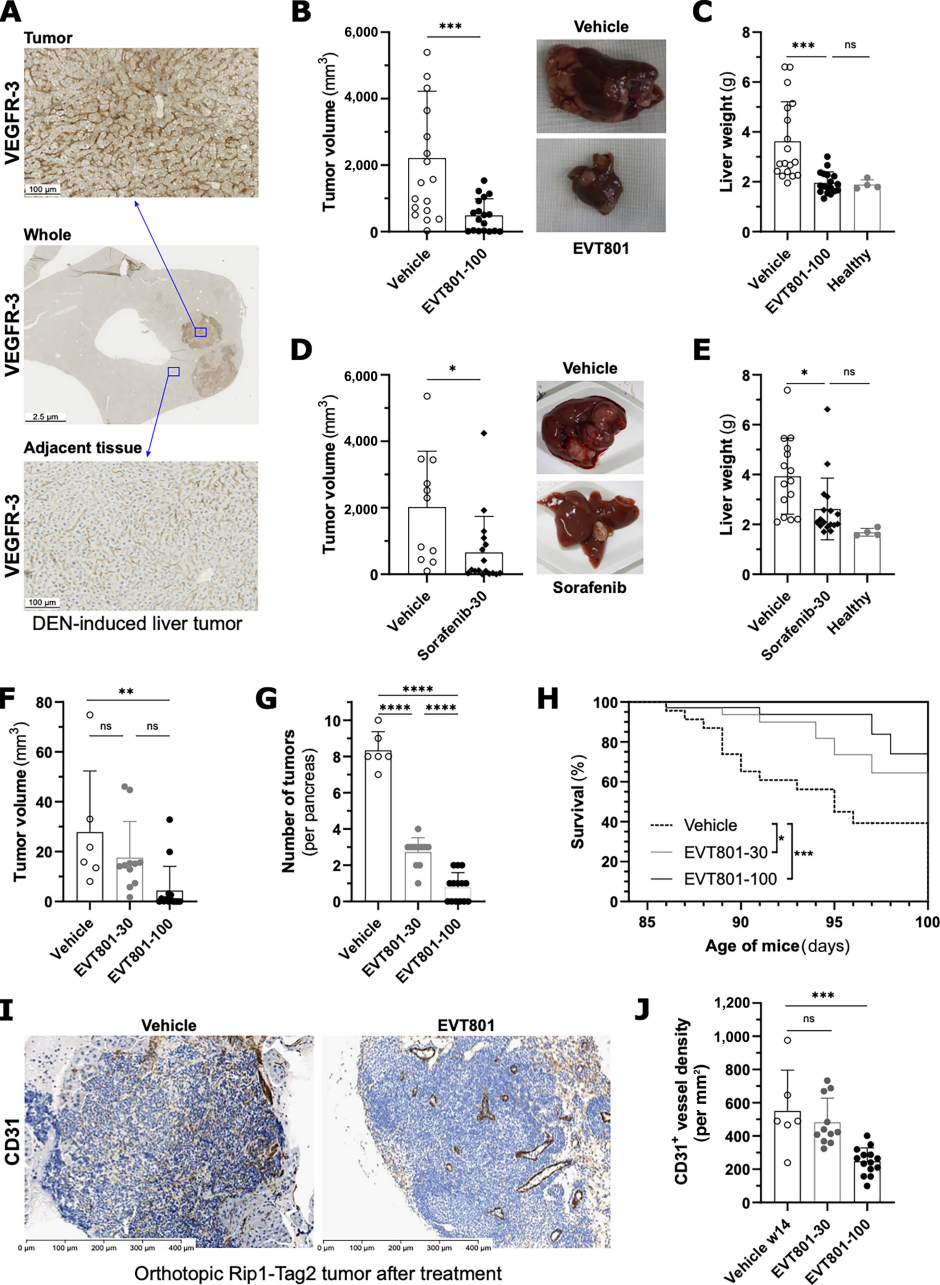
EVT801 inhibits proliferation and growth of tumors with VEGFR-3–positive TME (**A**). Representative IHC images of VEGFR-3 expression in liver tumors 12 months after DEN treatment. Macroscopic view of tumor within the liver (middle). Zoom on tumor (top) and adjacent normal tissue (bottom). **B,** Tumor volume after 2 months of daily treatment of DEN-induced liver tumor mouse model with vehicle or 100 mg/kg EVT801. Images on the right show representative whole liver after treatment. Nonparametric two-tailed Mann–Whitney test was used for statistical evaluation. **C,** Liver weight from DEN-induced liver tumor mouse model at endpoint after treatment with vehicle or 100 mg/kg EVT801. **D,** Tumor volume after 2 months of daily treatment of DEN-induced liver tumor mouse model with vehicle or 30 mg/kg sorafenib. Images on the right show representative whole liver after treatment. **E,** Liver weight of DEN-induced liver tumor mouse model at endpoint after treatment with vehicle or sorafenib. **F,** Orthotopic Rip1-Tag2 tumor volume after 2 weeks of daily treatment with vehicle, 30 mg/kg EVT801 or 100 mg/kg EVT801. **G,** Number of Rip1-Tag2 tumors per pancreas after 2 weeks of daily treatment with vehicle, 30 mg/kg EVT801 or 100 mg/kg EVT801. **H,** Survival of mice with orthotopic Rip1-Tag2 tumors upon treatment with vehicle, 30 mg/kg EVT801 or 100 mg/kg EVT801. **I,** Representative IHC images of tumor blood vessels by CD31 staining in orthotopic Rip1-Tag2 tumors upon treatment with vehicle or EVT801. **J,** Quantitation of intratumor vascular density in orthotopic Rip1-Tag2 tumors upon treatment vehicle, 30 mg/kg EVT801 or 100 mg/kg EVT801.

After demonstrating the effect of EVT801 on VEGFR-3–positive tumors, we explored how EVT801 can affect tumor growth when VEGFR-3 expression is restricted to the TME (as reported in Fig. 2). Because hepatocellular carcinoma is a major indication for the administration of VEGFR RTK inhibitors, we selected a DEN-induced liver tumor mouse model to further evaluate EVT801. Twelve months after DEN treatment, IHC imaging indicated that expression of VEGFR-3 in the tumor was localized in sinusoids and tumor blood vessels (Fig. 4A). Following the confirmation that VEGFR-3 was not expressed in tumor cells, treatment of mice started at month 12 with daily doses of 100 mg/kg EVT801 for 2 months. Compared with the vehicle-treated group, EVT801 substantially decreased tumor volume (T/C ratio of 22%; Fig. 4B). Furthermore, EVT801 fully prevented the increase in liver weight associated with tumor development, so that livers from EVT801-treated mice weighted like healthy mice livers (Fig. 4C). In addition, we compared the treatment outcome of EVT801 with sorafenib in subsequent experiments. Similar to EVT801, 30 mg/kg sorafenib decreased, tumor growth (T/C ratio of 32%; Fig. 4D), and reduced liver weight (Fig. 4E).

We then examined whether EVT801 impaired carcinogenesis using the Rip1-Tag2 transgenic mouse model of β-cell carcinogenesis, which we used earlier to evaluate SAR131675 (26), and which expresses VEGFR-3 only in the TME (42). We treated the mice with two different daily dose regimens of EVT801 (i.e., 30 and 100 mg/kg/day) over the course of 2 weeks. Of note, the higher dose of 100 mg/kg/day achieved the same plasma exposure as 30 mg/kg twice per day used in other models and was used for practical reasons (*C*_max_ equals 8,940 vs. 8,390 ng/mL; AUC_(0–24)_ equals 22,000 vs. 28,000 ng h mL^−1^). We compared the weight of EVT801-treated tumors with the ones of vehicle-treated tumors (week 14) and tumors before treatment (week 12). The lower dose of EVT801 led to a reduction of 37% in tumor volume (T/C ratio of 63%), while the higher dose of EVT801 reached a T/C ratio of 16% (Fig. 4F). In addition, EVT801 substantially and dose-dependently reduced the number of pancreatic tumors. The dose of 30 mg/kg/day yielded an average of three tumors per pancreas, corresponding to a reduction by 67%, whereas the dose of 100 mg/kg/day achieved 91% reduction, only leaving the primary tumor (Fig. 4G). Furthermore, EVT801 treatment dose-dependently increased the overall survival of the mice (Fig. 4H). We subsequently performed IHC labeling for CD31 and could evidence that EVT801 impacts density of CD31-expressing vessels, essentially leaving behind larger vessels (Fig. 4I), which was confirmed by the quantification (Fig. 4J).

### EVT801 Limits Hypoxia-driven Immunosuppression and Increases Anticancer Activity of Immune Checkpoint Inhibitors

**FIGURE 5:**
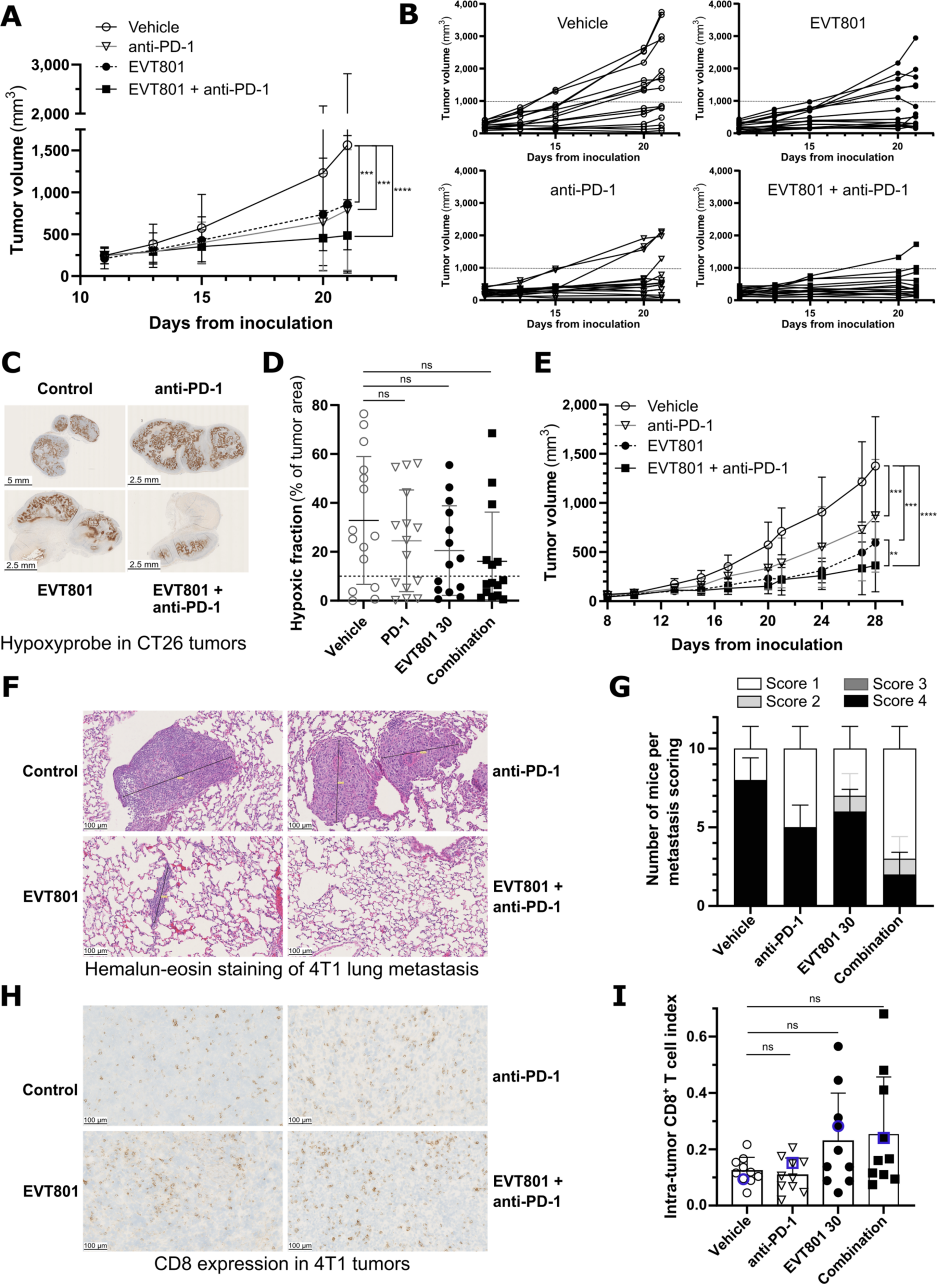
EVT801 blocks hypoxia-driven immunosuppression and enhances ICT efficacy. **A,** Tumor volume progression over time of subcutaneous CT26 tumors in BALB/c mice in presence of vehicle, anti-PD-1 (twice per week), 30 mg/kg EVT801 (twice per day) or a combination of anti-PD-1 and EVT801 (15 mice per group). **B,** Individual CT26 tumors in BALB/c mice for each of the four treatment groups. **C,** Representative IHC images of hypoxic zones (via hypoxyprobe) in CT26 tumors at endpoint after treatment with vehicle, anti-PD-1, EVT801, or both anti-PD-1 and EVT801. **D,** Hypoxyprobe signal in individual tumors at endpoint for each of the four treatment groups. **E,** Tumor volume progression over time of orthotopic 4T1 tumors in BALB/c mice in presence of vehicle, anti-PD-1 (twice per week), 30 mg/kg EVT801 (twice per day) or a combination of anti-PD-1 and EVT801 (15 mice per group). **F,** Representative hematoxylin-eosin staining of 4T1 lung metastasis at endpoint for each of the four treatment groups. **G,** Quantitation of lung metastasis in individual mice at endpoint for each of the four treatment groups. **H,** Representative IHC images of CD8^+^ T cells via CD8 expression in 4T1 tumors at endpoint for each of the four treatment groups. **I,** Frequency of CD8^+^ T cells in 4T1 tumors at endpoint for each of the four treatment groups. The highlighted datapoint (in blue) refers to the samples that was used for IHC imaging in **H**.

Our findings indicated that EVT801 decreased tumor angiogenesis without inducing hypoxia in the TME. Because hypoxia interferes with the immune system, we asked whether EVT801 limited these negative effects on the antitumor immunity and could advantageously combine with immunotherapy. First, we treated BALB/c mice carrying subcutaneous CT26 tumors either with an anti-PD-1 mAb (anti-PD-1), with 30 mg/kg twice per day EVT801, or with a combination thereof (Fig. 5A). As expected, anti-PD-1 significantly reduced tumor volume compared with the control group (T/C ratio of 51%). EVT801 also showed a significant effect (T/C ratio of 55%) which was not as pronounced as in the previously described models, consistently with the lower expression level of VEGFR-3 in this tumor model. Remarkably, the combination of anti-PD-1 and EVT801 yielded a substantial decrease in tumor volume (T/C ratio of 31%). Analysis of individual tumor volume progression over time showed heterogeneity in the response to ICT treatment, indicated by responder and nonresponder mice, whereas the combination of EVT801 and anti-PD-1 yielded a more homogenous effect on tumor growth because all but one tumor responded to treatment (Fig. 5B). At the end of the treatment, we performed IHC on the CT26 tumors using anti-pimonidazole (i.e., hypoxyprobe) to assess the hypoxic state (Fig. 5C). Quantitation of the hypoxyprobe-positive areas showed a reduction of 25% with anti-PD-1, 42% with EVT801, and 51% with the combination compared with vehicle (Fig. 5D). The observed reduction in hypoxic zones was not statistically significant due to very high interindividual variability; nevertheless, the number of mice with hypoxia-free tumors in EVT801-treated and combination-treated groups was higher than in the control group. Of note, the observed effect on the hypoxic zones upon EVT801 treatment was in agreement with the observation in BNL-R3 tumor mouse models (as reported in Supplementary Fig. S2D).

To determine whether EVT801 supported immunotherapy in a metastatic mouse model, we orthotopically injected 4T1 tumors in BALB/c mice, which showed an intermediate expression of VEGFR-3 in the TME. Mice were treated for 2 weeks with 30 mg/kg twice per day EVT801 and/or anti-PD-1 twice per week (Fig. 5E). 4T1 tumor volume was significantly reduced by EVT801 and anti-PD-1 (T/C ratio of 43% and 63%, respectively), whereas the combination of both compounds led to larger reduction (T/C ratio of 31%). Subsequent hematoxylin-eosin staining of the 4T1 lung metastasis from the different treatment groups showed a reduction in size and number of metastasis, which corresponds to the decrease in tumor volume progression (Fig. 5F). This observation was confirmed by individual scoring using a composite score, taking into account number and size of metastasis (Fig. 5G). We finally assessed the potential effect of the treatments on infiltration of CD8^+^ T cells in 4T1 tumors. IHC imaging revealed increased CD8 expression in some tumors upon treatment with EVT801 alone or in combination with anti-PD-1 (Fig. 5H). Quantification of CD8 staining in tumors confirmed that anti-PD-1 did not lead to enrichment of the intratumor CD8^+^ T-cell population, whereas EVT801 alone and in combination with anti-PD-1 globally doubled the number of infiltrated CD8^+^ cells; however, the analysis did not achieve statistical significance due to high interindividual variability (Fig. 5I).

### EVT801 Reduces Circulation of Myelosuppressive Chemokines and MDSCs in the Blood

**FIGURE 6:**
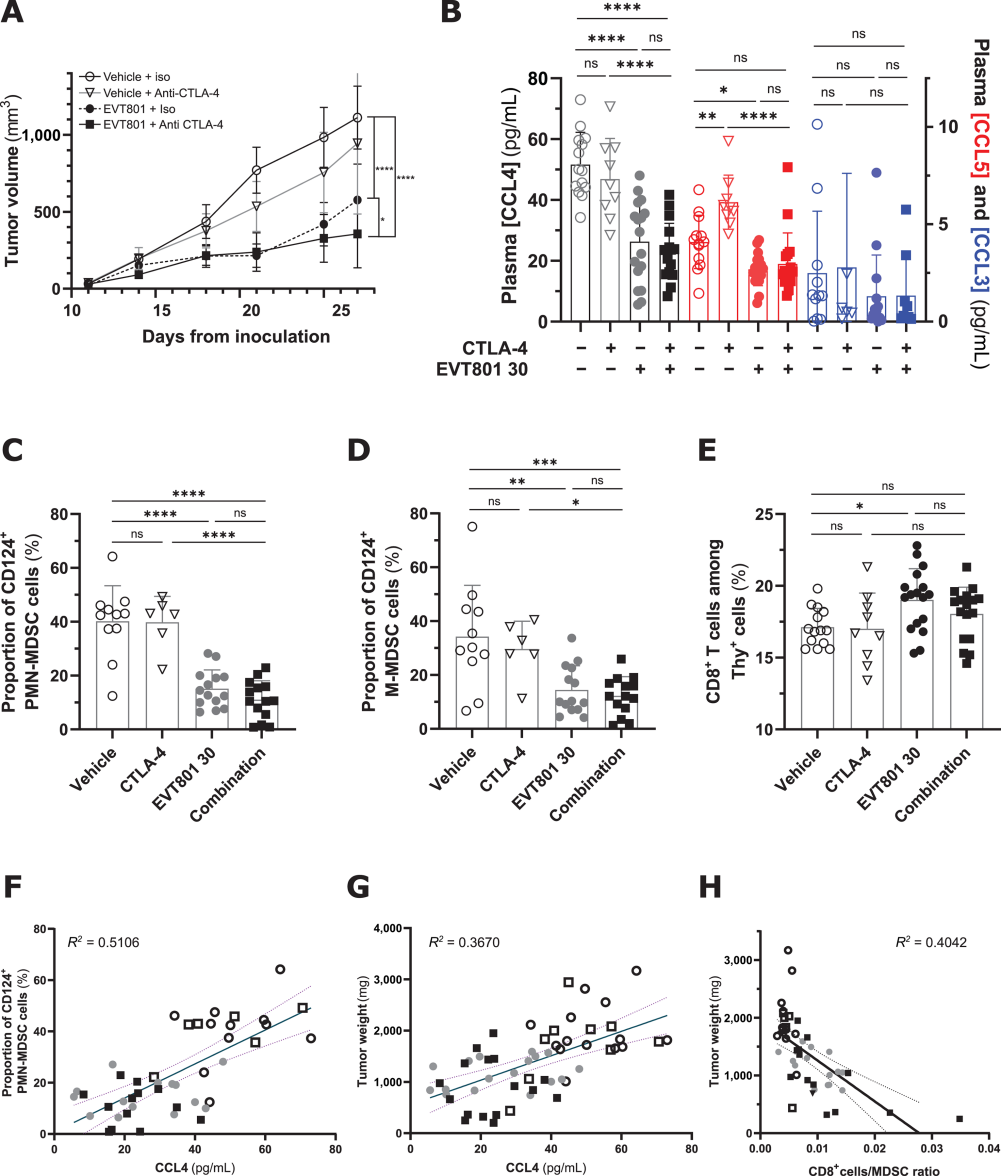
EVT801 reduces circulation of chemokines and MDSCs in the blood. **A,** Tumor volume progression over time of orthotopic 4T1 tumors in BALB/c mice in presence of vehicle, isotype control, anti-CTLA-4 (twice per week), 30 mg/kg EVT801 (twice per day), or a combination of anti-PD-1 and EVT801. **B,** Individual plasma concentrations of CCL3/MIP-1α, CCL4/MIP-1β, and CCL5/Rantes at endpoint for each of the four treatment groups. **C,** Individual blood concentration of CD124^+^ PMN-MDSCs at endpoint for each of the four treatment groups. **D,** Individual blood concentration of CD124^+^ M-MDSCs at endpoint for each of the four treatment groups. **E,** Individual blood concentration of CD8^+^ T cells at endpoint for each of the four treatment groups. **F,** Correlation between circulating CCL4 and PMN-MDSC levels. **G,** Correlation between circulating CCL4 and tumor weight. **H,** Inverse correlation between CD8^+^ T-cell/PMN-MDSC ratio and tumor weight.

To get a better view of the chronology and interdependence of EVT801 effects between inhibition of (lymph)angiogenesis and T-cell infiltration, we decided to explore further EVT801’s systemic effect on the immune system. We decided to switch to an anti-CTLA-4 mAb (anti-CTLA-4) because this ICT is considered to affect the early T-cell immune response in lymph nodes, whereas anti-PD-1 suppresses the late T-cell response in peripheral tissues (43, 44). When the orthotopic 4T1 tumors were measurable (20–40 mm^3^), we started treatment and tumor volume monitoring in different groups that received either (i) vehicle and isotype control, (ii) 30 mg/kg twice per day EVT801, (iii) anti-CTLA-4 twice per week, or (iv) a combination of EVT801 and anti-CTLA-4 (Fig. 6A). Anti-CTLA-4 showed no significant effect, whereas EVT801 alone and the combination of both demonstrated significant inhibition of tumor growth (T/C ratios of 53 and 32%, respectively). We examined at endpoint the myelosuppressive chemokines CCL3/MIP-1α, CCL4/MIP-1β and CCL5/Rantes, which are known to play a crucial role in proliferation, activation, and mobilization of MDSCs from the bone marrow to the blood (45). We found that 30 mg/kg twice per day EVT801 yielded significant decrease in circulating CCL5 and CCL4 levels (−30% and −50%), whereas CCL3 levels were unchanged (Fig. 6B).

In addition to cytokines, we investigated the impact of EVT801 on circulating immune cells, in particular MDSCs and CD8^+^ T cells. MDSCs expand during cancer and regulate immune responses by suppressing T-cell activity so they are crucial biomarkers for antitumor immunity (41, 46). Hence, we employed flow cytometry to quantify the circulating activated (CD124^+^) PMN-MDSCs and M-MDSCs. We observed 14 hours after the last dosing a 2-fold reduction in the circulation of each MDSC subpopulation in the blood (Fig. 6C and D) in mice treated with EVT801 alone or combined with anti-CTLA-4. In addition, the analysis demonstrated that EVT801 induced a 30% increase in the number of circulating CD8^+^ T cells (Fig. 6E). Moreover, the data showed a correlation between CCL4 levels and number of PMN-MDSCs in circulation (*R*^2^ = 0.5106) as well as tumor weight (*R*^2^ = 0.3670; Fig. 6F and G). Furthermore, the CD8^+^ T-cell/PMN-MDSC ratio showed an inverse correlation with tumor weight (*R*^2^ = −0.4042; Fig. 6H), which altogether could have important ramification for clinically relevant biomarker considerations.

## Discussion

As of today, a number of angiogenesis RTK inhibitors are approved for the treatment of cancer; nonetheless, they are not selective, and thus affect the TME predominantly by shrinking vasculature, which worsens hypoxia. A growing body of evidence shows that hypoxia promotes tumorigenesis and, furthermore, confers drug resistance by altering the tumor cell physiology (47). Moreover, the currently available RTK inhibitors are associated with considerable adverse effects, such as hypertension, anorexia, and fatigue, which are due to their broad target interaction profiles. The development of more selective inhibitors promises lower hypoxia-induced resistance, less tumor escape via lymphatic vessels and fewer side effects than the current generation of inhibitors.

Here, we present EVT801, a selective, orally available VEGFR-3 inhibitor, developed for the treatment of solid tumors. EVT801 demonstrated strong antitumor effects in various *in vitro* and *in vivo* models, including the 4T1 and the Rip1-Tag2 mouse models, which were used to evaluate the earlier described compound SAR131675 (26). Interestingly, EVT801 *in vivo* efficacy was at least as good as the pleiotropic inhibitors sorafenib and pazopanib. This supports the approach of selective VEGFR-3 pathway inhibition to achieve antitumor effects by cooperative modulation of lymphatic and blood vessels in the tumor, which suppresses VEGFR-3–mediated immune tolerance and prevents macrophage polarization into immunosuppressive cells (28). Moreover, EVT801 did not increase blood pressure at a dose as high as 500 mg/kg in telemetered rat model of hypertension, whereas sorafenib showed the opposite effect (Supplementary Fig. S8). The same behavior was observed for both compounds in telemetered monkeys during regulatory toxicology studies.

An earlier study showed that VEGFR-3 signaling is critical during embryonic vasculogenesis (48). Moreover, it was also reported that inhibition of the VEGFR-3 pathway impairs angiogenesis, halts tumor development, and leads to reduction of hypoxia in the TME (49). In agreement with those findings, we demonstrated that EVT801 reduced tumor (lymph)angiogenesis and, apparently, impacted preferentially small tumor vessels over larger ones. This finding implies that the observed reduction of tumor hypoxia was a consequence of the impact of EVT801 on the tumor vasculature, whereas sorafenib did not produce such level of reduction in hypoxia (50). Abating hypoxia in the TME also reduces the immunosuppressive effect, yielding an enhanced immune response (51, 52). This is highlighted by the reduction in circulating MDSCs and the chemokines CCL4 and CCL5 upon EVT801 administration. Furthermore, this also contributed to increased CD8^+^ T-cell infiltration in the orthotopic 4T1 tumor mouse model and similar effects on MDSCs and CD3^+^ and CD8^+^ cells in the BNL-R3 subcutaneous model, further supporting the contribution of EVT801 to anticancer immunity. Of note, we could not detect VEGFR-3 in human MDSCs from a small cohort of patients, thus we believe that the observed effect on MDSCs is an indirect consequence of EVT801 treatment. Further studies are required to obtain a comprehensive view of the underlying mechanism, and eventually find the best combination options. For instance, interrogating the TME and expanding chemokine measurement to also include the proinflammatory CXCL9-11 and immunosuppressive CXCL12 or CCL22 may provide important insights on the nature of EVT801 anticancer effect. Another important consideration is the role of VEGFR-3 in lymphangiogenesis (53). Formation of new lymphatic vessels allows tumor cells to spread to distant sites (54). Hence, impairment of lymphangiogenesis results in the reduction of metastasis (55). Indeed, our findings in kidney cancer samples and in the 4T1 mouse model support that inhibition of VEGFR-3 by EVT801 reduces metastasis. Nonetheless, additional data are required to demonstrate the effect of EVT801 on tumor lymphangiogenesis.

We have shown that EVT801 targets the VEGFR-3 pathway, and thus affects VEGFR-3–positive tumors and tumors with a VEGFR-3–positive TME. The limited effect as single agent observed in the CT26 model suggests that EVT801 efficacy is linked to VEGFR-3 expression levels. This begs the question whether there is a minimal level of VEGFR-3 expression that is required for EVT801 to produce an effect. Our experiments on the patient-derived xenograft tumor model RT-HAM-001 demonstrated that a significant effect can be obtained in a model in which only 20% of tumor cells express VEGFR-3. Nevertheless, in this particular model, expression of mouse VEGFR-3 in the TME was not assessed, and EVT801 activity is likely due to inhibition of VEGFR-3 in both tumor and TME. Hence, it will be important in future studies in patients to take into account both tumor and TME to determine the minimum threshold of VEGFR-3 expression for effective EVT801 application. This would have important ramifications for patient stratification in clinical studies, and eventually for the success of EVT801 throughout clinical development.

Taken together, our research demonstrates a novel anti(lymph)angiogenic compound that selectively targets VEGFR-3, modulates the TME to induce tumor blood vessel homogenization (i.e., leaving fewer and overall larger vessels), and enhances immunotherapy. The proposed mode of action of EVT801 encompasses three consecutive anticancer mechanisms, which all contribute to inhibition of tumor growth and metastasis (Supplementary Fig. S9). First, the selective inhibition of VEGFR-3 by EVT801 prevents tumor growth by impairing both tumor angiogenesis and lymphangiogenesis, which stabilizes tumor vasculature, decreases metastasis, and reduces hypoxia in the TME. Second, abating hypoxia strengthens anticancer immunity, reflected by a decrease in immunosuppressive cytokines and cells (i.e., MDSCs) in circulation and tumor surroundings. Third, this promotes T-cell infiltration in the tumor, eventually supporting an increased and long-term antitumor immune response. Of note, the effect of EVT801 on lymphangiogenesis is in agreement with the reported role of VEGFR-3 in the lymphatic system; nonetheless, this effect requires experimental validation because the methods used in this study do not discriminate between blood and lymph vessels.

On the basis of these promising results, EVT801 was selected to enter clinical trials as a highly selective VEGFR-3 modulator, targeting tumor angiogenesis and lymphangiogenesis in patients. EVT801 is evaluated as single agent (NCT05114668), and combination with cancer immunotherapies will come next

## Supplementary Material

Supplementary Materials and Methods SM1 supplementary materials & methods

Supplementary Figure S1 Supplementary Figure S1 shows that EVT801 metabolite is a selective VEGFR-3 inhibitor

Supplementary Figure S2 Supplementary Figure S2 shows that EVT801 inhibits tubule formation

Supplementary Figure S3 Supplementary Figure S3 shows the expression of VEGFR3 altogether with vascular and lymphatic markers in primary kidney tumors

Supplementary Figure S4 Supplementary Figure S4 shows the evaluation of EVT801’s pharmacokinetics and pharmacodynamics

Supplementary Figure S5 Supplementary Figure S5 shows the immunohistochemical evaluation of hypoxia in NCI-H1703 subcutaneous tumor model

Supplementary Figure S6 Supplementary Figure S6 shows that EVT801 inhibits growth of VEGFR3-positive BNL hepatoma tumors, while decreasing vasculature and hypoxia

Supplementary Figure S7 Supplementary Figure S7 shows that EVT801 decreases immunosuppressive cell infiltration in VEGFR3-positive BNL hepatoma tumors

Supplementary Figure S8 Supplementary Figure S8 shows that EVT801 doesn’t modify blood pressure in rat while sorafenib increases it

Supplementary Figure S9 Supplementary Figure S9 provides an overview of the mechanisms by which EVT801 exerts its anti-tumor effects

Supplementary Table TS1 Supplementary Table S1 shows EVT801 potency and selectivity profile

Supplementary Table TS2 Supplementary Table S2 shows EVT801 metabolite potency and selectivity profile

Supplementary Table TS3 Supplementary Table S3 shows pharmacokinetic parameters of EVT801 in mice and rats
