# Supplementary material for: Targeting Tumor Angiogenesis with the Selective VEGFR-3 Inhibitor EVT801 in Combination with Cancer Immunotherapy
Source: Cancer Res Commun. 2022 Nov 29;2(11):1504–19. doi: 10.1158/2767-9764.CRC-22-0151 (PMC10035370; doi:10.1158/2767-9764.CRC-22-0151)
Supplement: Supplementary Materials and Methods SM1 — supplementary materials & methods [file crc-22-0151-s01.docx]

**SUPPLEMENTARY MATERIAL AND METHODS**

**Kinase panel**

Recombinant human proteins from different vendors (Carna Bio, Millipore, Invitrogen) were suspended in phosphate buffer, pH 7.4 containing 1 mM DTT and pre-incubated with EVT801 or SAR401849 for 15 min. Then, substrate and ATP were added (final concentrations of 1.5 µM and equal to K_m_), the mixture incubated for 90 min at 28 °C. After incubation, 25 µL ADP-Glo reagent (Promega) was added to the reactions, and incubated at room temperature for 40 min. Then, 50 µL of kinase detection reagent was added and incubated for 40 min. Subsequently, the reaction was stopped with EDTA, and luminescence was recorded and IC_50_ values were determined.

**Biochemical kinase assay**

Sigma protein kinase assay kits were used. 125 μL per well of Poly-Glu-Tyr (PGT) solution (250 μg/mL of PGT in PBS without Ca^2+^, Mg^2+^ and sodium bicarbonate) was added in a 96-well plate and overnight incubation was performed at 37 °C. The day after, coating solution was removed and wells were washed with 300 μL of PBS-Tween-20 0.05 %. The washing buffer was removed and wells were dried for 2 h at 37 °C. 90 μL of the kinase buffer was supplemented with ATP (30 μM for VEGFR-1 and VEGFR-3, and 15 μM for VEGFR-2), and the compound (EVT801 or SAR401849 at the indicated concentration or 1 % DMSO for the vehicle control). The phosphorylation reaction was initiated by the addition of 20 μL of kinase buffer without ATP supplemented with 100 ng of the tyrosine kinase (or without tyrosine kinase as control). Plates were incubated with gentle agitation at room temperature for 30 min. After 3 washes with 300 μL of washing buffer, 100 μL of phosphotyrosine specific monoclonal antibody conjugated to HRP (1:30,000) was added onto the phosphorylated PGT. Plates were incubated for 30 min at room temperature under gentle agitation. Then, 3 washes with 300 μL of washing buffer were performed. 100 μL of the HRP chromogenic substrate (SIGMAFAST™ OPD, Sigma-Aldrich) was added in the dark. After 7 min at room temperature, the reaction was stopped by addition of 100 μL of 1.25 μM H_2_SO_4_, and absorbance was determined with an Endvision spectrophotometer at 492 nm.

**VEGFR inhibition *in cellulo***

Transfection of HEK293T cells and Ba/F3 cells with VEGFR-1-Flag, VEGFR-2-Flag or VEGFR-3-Flag expression constructs was performed using Fugene-6 (Roche) as per manufacturer’s protocol. Briefly, Fugene-6 was pre-incubated with Opti-Mem (Gibco) before adding DNA. After 24 h, media was replaced with serum-free culture media containing 0.2 % BSA. On the next day, cells were incubated for 1 h in 1 mM orthovanadate, harvested, dissociated and distributed at 4 x 10^5^ cells in 5 mL for HEK-VEGFR-1 and HEK-VEGFR-3, and 5 x 10^4^ cells per 5 mL for HEK VEGFR-2 in the presence of EVT801 or SAR401849. After 30 min, the reaction was stopped by the addition of cold PBS supplemented with 1 mM orthovanadate. Cells were then lysed with 150 μl of RIPA buffer for 15 min at 4°C, then centrifuged for 10 min at 10,000 g. Supernatants (75 μl) were distributed in duplicate on 96 well plates that were precoated with the anti-flag, and left for 1 h at room temperature. After 3 washes, the anti-phosphotyrosine conjugated to the HRP was added and incubated for 1 h at room temperature. Wells were then washed 3 times with TBS buffer containing 0.5 % Tween-20 and 2 mM MgCl_2_. The reaction was stopped with 50 μL of 2 N H_2_SO_4_, and the signal was read with Envision at the wavelengths of 485 nm and 530 nm.

Ba/F3 cells were transduced and selected to stably express fusion proteins consisting of constructs for IL-3 independent survival and either VEGFR-2 or VEGFR-3 kinase domains or without the kinase domain. Proliferation and survival of cell lines was dependent on expression of the transcript. Cells in logarithmic-phase growth were harvested and 5 x 10^3^ cells were distributed into each well of a 384-well plate in 50 μL of medium. Then, 50 nL of EVT801, SAR401849 or their vehicle solvent were added to appropriate wells. After 48 h, viability was determined by addition of 10 μL CellTiterGlo reagent (Promega). Luminescence was determined as relative light units (RLU) in counts per second.

**Detection of ERK phosphorylation**

hLMVECs were plated in 6-well tissue culture plates at a density of 2 x 10^5^ cells for 48 h. Cells were then serum deprived for 2 h and either stimulated or mock stimulated for 10 min with VEGFC (500 ng/mL) in the presence or absence of EVT801. Then, cells were lysed and subjected to phospho-MAPK array or to phospho-ERK ELISA according to manufacturer’s instructions.

For immunoblotting, 2 x 10^5^ cells were scraped and lysed with RIPA lysis buffer (Sigma, R0278), supplemented with protease inhibitor cocktail (Ozyme, 5872S). After separation by SDS-PAGE, proteins were transferred to PVDF membranes. Membranes were blocked with 5 % milk in TBS-T for saturation step and incubated with phosphorylated ERK (CellSignaling, 4377, 1:1000) or GAPDH (CellSignaling, 5174, 1:1000) antibodies overnight, followed by an anti-mouse IgG, secondary antibody HRP linked (CellSignaling, 7076, 1:2000) in antibodies buffer (TBS-Tween 0.05 %, 0.1 % BSA) for 2 h. Membranes were washed 3 x 10 min in TBS before adding chemiluminescent substrate (Supersignal West Pico Plus, 54580, ThermoFisher) and imaged by the Pxi6 station (Ozyme).

**Flow cytometry of VEGFR-3**

For VEGFR-3 staining, 2 x 10^5^ hLMVEC or NCI-H1703 cells were washed in PBS and suspended in a final volume of 100 µl per tube. Cells were incubated for 30 min at 4°C in the dark with anti-VEGFR-3 (Millipore, MAB3757, 1:50) and the LIVE/DEAD Fixable Aqua Dead Cell Stain kit (Molecular Probes™, 15511863, Thermo Fisher Scientific, 1:300). Then, cells were washed with PBS. After resuspension in 100 µl PBS, cells were incubated with the Mouse F(ab)2 IgG (H+L) PE-conjugated antibody (R&D Systems, F0102B, 1:50) for 30 min at 4°C in the dark. After washing with PBS, cells were suspended in 300 µl PBS and analyzed by flow cytometry (Fortessa X20, BD). Acquisition and analysis were performed via BD FACSDiva Software.

**Cell viability and proliferation assays**

NCI-H1703 were seeded in 96-well plates coated with 0.3 % gelatin (5 x 10^3^ cells per well) and were incubated in RPMI 0.1 % FCS with VEGF-C (300 ng/mL) or VEGF-D (300 ng/mL) in the absence or presence of increasing doses of EVT801. After 5 days, viable cells were quantified with the cell Titer-glo luminescent cell viability assay (Promega) as previously described (1).

hLMVEC were seeded in white cell culture-treated flat and clear bottom multi-well plates and incubated at 37 °C overnight, before compounds were added. After incubation for 72 h at 37 °C, plates were equilibrated to ambient temperature for 1 h, and CellTiterGlo reagent (Promega) was added. After approximately 1h, luminescence was measured using a luminometer. Curves were fitted via sigmoidal dose response curves, and IC_50_ values were determined.

**Aortic ring assay**

Neo-angiogenesis was studied *in vitro* by culturing rings of mouse thoracic aorta in 3-dimensional collagen gels, as previously described (2), but the mouse serum was replaced by a piece of 4T1 tumor. Briefly, 1 mm-long aortic ring-shaped fragments of 9-week old C57Bl/6J mice, and 1 mm^3^ 4T1 tumor pieces resected 21 days after orthotopic injection in mammary fat pad of BALB/c mice were embedded in a rat tail interstitial collagen gel (1.5 mg/mL). It was prepared by mixing 7.5 volumes of 2 mg/mL collagen (Collagen R, Serva), 1 volume of 10 x minimum essential medium (MEM, Life Technologies Ltd.), 1.5 volumes of NaHCO_3_ (15.6 mg/mL), and 0.1 volume 1 M NaOH to adjust the pH to 7.4. The collagen gels containing the aortic rings were polymerized in cylindrical agarose wells, and kept in triplicate at 37 °C in 60 mm diameter Petri dishes (bacteriological polystyrene; Falcon, Becton Dickinson). Each dish contained 6 mL of MCDB131 (Life Technologies Ltd.) supplemented with 25 mM NaHCO_3_, 1 % glutamine, 100 U/mL penicillin, and 100 µg/mL streptomycin. The cultures were kept at 37 °C in a humidified environment for 8 days and examined every other day with a Nikon microscope at appropriate magnification. EVT801 (300 nM) or control vehicle was added in the culture medium at day 0 and day 3. Quantification of pseudo-tubules was performed at day 8 using an automated computer-assisted image analysis system (Morpho Expert V2.50, Explora Nova, La Rochelle, France). Experiments were performed in triplicate.

**PK and formulation of compounds**

Plasma samples and tumor homogenates were mixed with the precipitant solution (acetonitrile containing internal standard). The supernatant (180 µL) was transferred in a 96-well plate and 5 µL were injected. EVT801 and its main metabolite were quantified by LC-MS/MS following standard conditions.

Quality control (QC) samples and calibration samples were prepared daily by spiking mouse plasma and tumor homogenates with working solutions prepared from independent weighing. The calibration curve was calculated from calibration levels at 1, 2.5, 5, 10, 25, 50, 100, 200, 500, 1000 and 2000 ng/mL. A variation of ± 20 % versus the nominal concentrations was used as criteria for qualification of samples; at least 75 % of all standards needed to be within this window, and at least 6 concentration values within tis window were used for quantification. The concentrations for QC samples were 5, 50, 150, 450 and 1500 ng/mL. At least 66 % of all QC samples, and at least 50 % of QC samples at a given concentration were within the ± 20 % window. Quadratic regressions were applied, not forced through the origin, and weighted by 1/x (with R^2^ > 0.98). The lower limit of quantification for both compounds was set to 1 ng/mL for plasma and to 3 ng/g for tumor samples.

For use in the NCI-H1703 tumor mouse model as well as in the RT-001-HAM PDx mouse model, EVT801 was formulated in a mixture of Soluplus (BASF), water, and hydroxypropylcellulose SL (Nisso America) in the ratio of 2:96:2 by continuous stirring at room temperature. EVT801 phosphate salt was solubilized in vehicle to a concentration of 3 mg/mL, and was administered to mice at a dose of 10 mL/kg. In the same tumor model, pazopanib was formulated as a suspension in HPMCT (0.5 %) + Tween-80 (0.1 %). There was no correction ratio for free base determination of drug. Pazopanib was solubilized in vehicle to a concentration of 3 mg/mL, and administered to mice at a dose of 10 mL/kg.

In RT-001-HAM PDx model, both EVT801 and pazopanib were formulated in (Soluplus (BASF)/water/hydroxypropylcellulose SL (Nisso America).

**VEGFR3 expressing BNL tumor model (BNL-R3)**

A total number of 60 female BALB/c mice were injected subcutaneously on the flank with 2 x 10^6^ cells in 250 μL PBS buffer/growth factor reduced Matrigel^®^. Mice were then kept in a post-operative cabinet at 28 °C until complete recovery. Assuming an ovoid form, tumor volume in mm3 was calculated using digital callipers according to the following formula: V = 0.52 x (width)² x length. On day 7, mice were randomized into 4 groups of 11 mice and 1 group with 16 mice, according to mean tumor volume via cage equalizer software. Subsequently, tumor volume was measured on days 11, 13, 15, 18, 19 and 21 following tumor cell injection. The selected groups received vehicle or EVT801 orally in a volume of 10 mL/kg. Mice body weight was measured daily before dosing. On the day of termination, mice were anaesthetized to facilitate 500 μL blood withdrawal by puncture of the jugular vein. Three mice per group and per time point (i.e., 0.5, 2, 4 and 24 h after the last dosing) were allocated for pharmacokinetic study. Spleens of 3 mice per group were collected, and immune cell populations were separated from dissociated splenocytes via MACS, and subjected to flow cytometry analysis.

**SUPPLEMENTARY REFERENCES**

1. Alam A, Blanc I, Gueguen-Dorbes G, Duclos O, Bonnin J, Barron P, et al. SAR131675, a potent and selective VEGFR-3-TK inhibitor with antilymphangiogenic, antitumoral, and antimetastatic activities. Mol Cancer Ther. 2012;11(8):1637–49.
2. Masson V, Devy L, Grignet-Debrus C, Bernt S, Bajou K, Blacher S, et al. Mouse aortic ring assay: A new approach of the molecular genetics of angiogenesis. Biol Proced Online. 2002 Jun;4(1):24.
