# Supplementary material for: Targeting Tumor Angiogenesis with the Selective VEGFR-3 Inhibitor EVT801 in Combination with Cancer Immunotherapy
Source: Cancer Res Commun. 2022 Nov 29;2(11):1504–19. doi: 10.1158/2767-9764.CRC-22-0151 (PMC10035370; doi:10.1158/2767-9764.CRC-22-0151)
Supplement: Supplementary Figure S1 — shows that EVT801 metabolite is a selective VEGFR-3 inhibitor [file crc-22-0151-s02.docx]

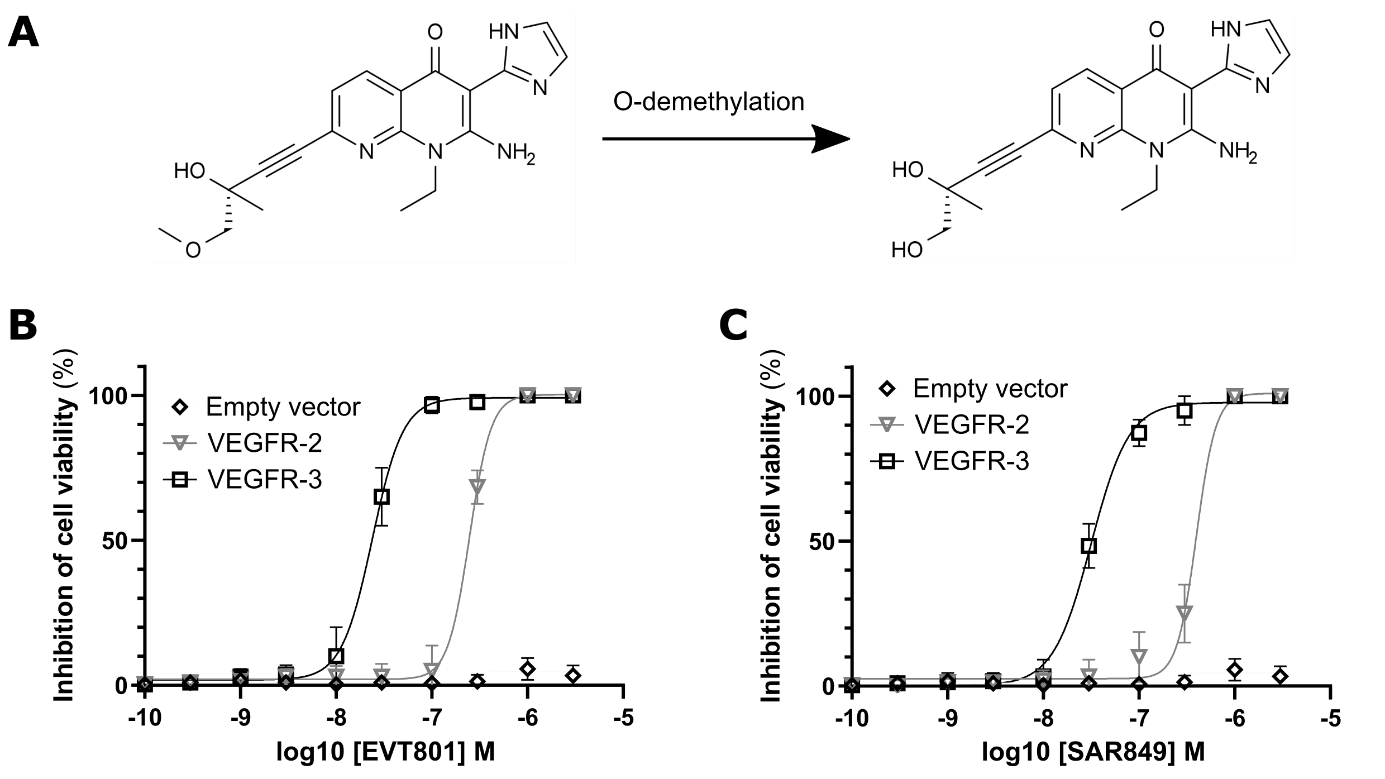


**Supplementary Figure 1.** EVT801 active metabolite is a selective VEGFR-3 inhibitor.

(A) Scheme of biotransformation of EVT801 into O-demethylated active metabolite SAR401849. (B) Dose response curves of the inhibition of VEGFR-2 and VEGFR-3 autophosphorylation by EVT801 in Ba/F3 cells. (C) Dose response curves of the inhibition of VEGFR-2 and VEGFR-3 autophosphorylation by SAR401849 in Ba/F3 cells.
