# Supplementary material for: Targeting Tumor Angiogenesis with the Selective VEGFR-3 Inhibitor EVT801 in Combination with Cancer Immunotherapy
Source: Cancer Res Commun. 2022 Nov 29;2(11):1504–19. doi: 10.1158/2767-9764.CRC-22-0151 (PMC10035370; doi:10.1158/2767-9764.CRC-22-0151)
Supplement: Supplementary Figure S2 — shows that EVT801 inhibits tubule formation [file crc-22-0151-s03.docx]

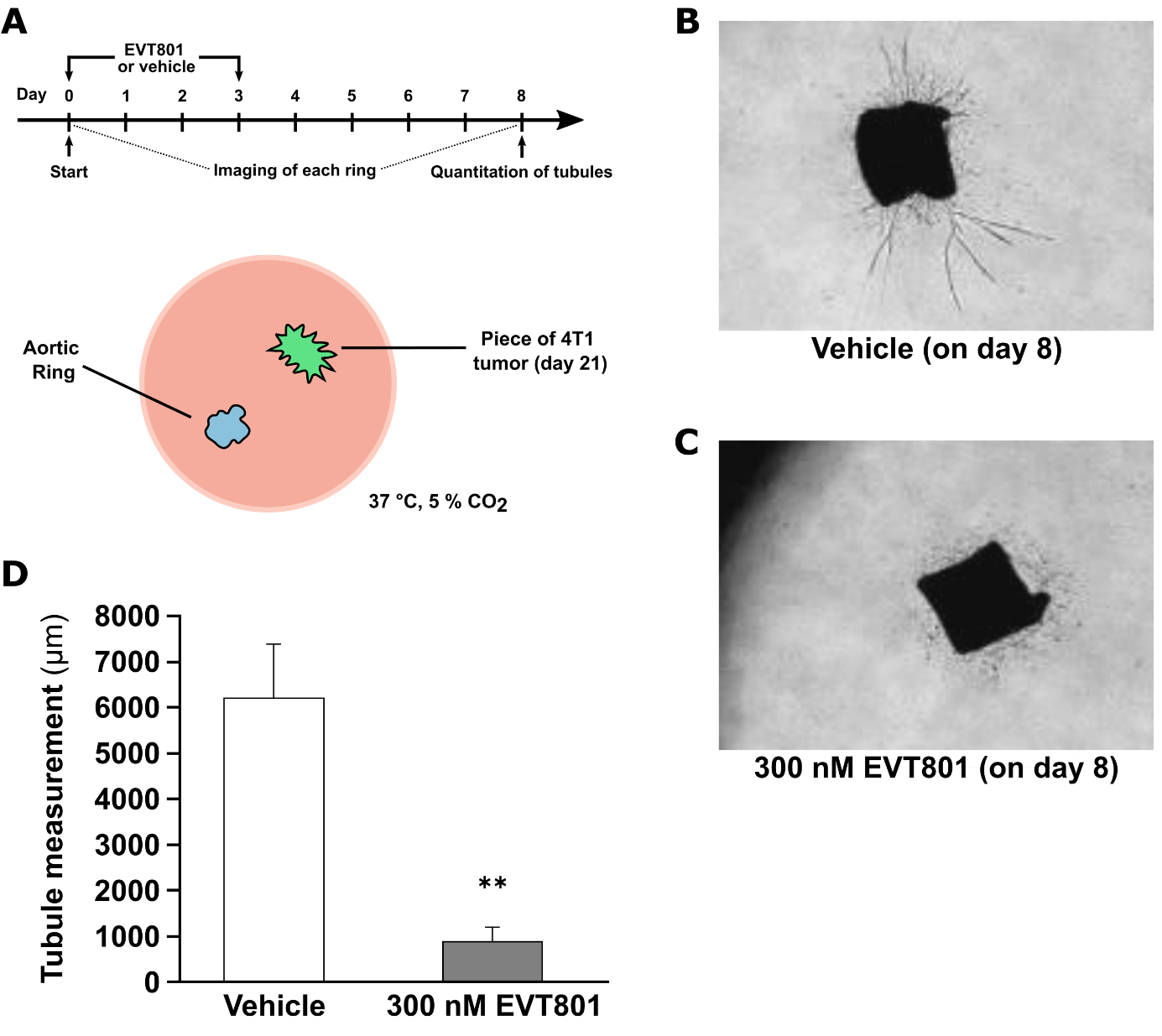


**Supplementary Figure 2.** EVT801 inhibits tubule formation in the mouse aortic ring assay stimulated by a 4T1 tumor fragment. Aortic rings (length of approx.1 mm) were cultured in the presence of 4T1 tumor fragment (approx. 1 mm^3^) for 8 days in medium containing either vehicle or 300 nM EVT801 (A). Representative images from aortic rings in presence of vehicle (B) or EVT801 (C) were taken at day 8. Quantitation of tubule length showed that EVT801 prevents tumor-induced tubule formation almost completely (D).
