# Supplementary material for: Targeting Tumor Angiogenesis with the Selective VEGFR-3 Inhibitor EVT801 in Combination with Cancer Immunotherapy
Source: Cancer Res Commun. 2022 Nov 29;2(11):1504–19. doi: 10.1158/2767-9764.CRC-22-0151 (PMC10035370; doi:10.1158/2767-9764.CRC-22-0151)
Supplement: Supplementary Figure S3 — shows the expression of VEGFR3 altogether with vascular and lymphatic markers in primary kidney tumors [file crc-22-0151-s04.docx]

**
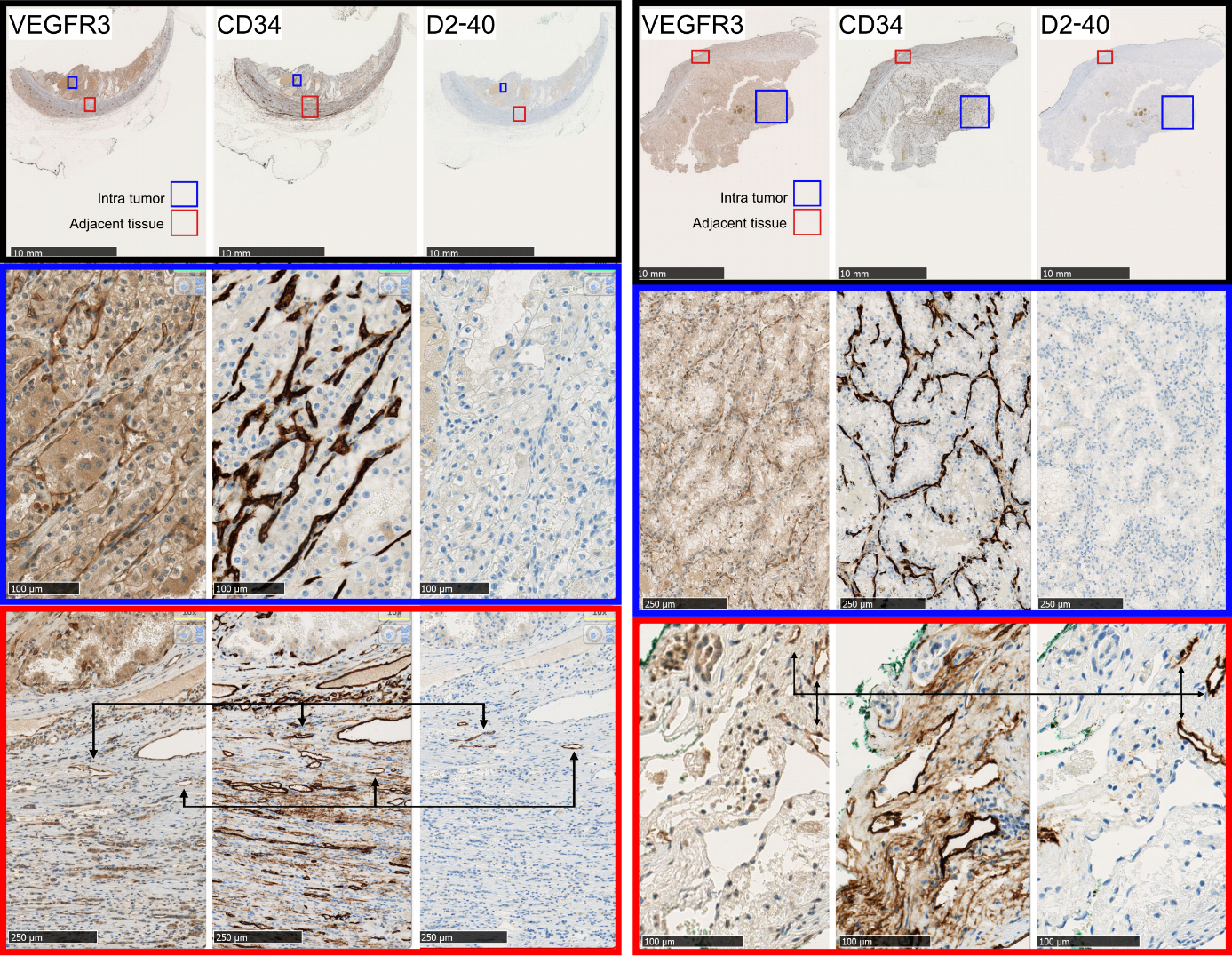
**

**Supplementary Figure 3.** Expression of vascular marker CD34, lymphatic marker D2-40 and VEGFR-3 in primary kidney tumors. Consecutive slices of the same tumor were stained for VEGFR-3, CD34 and D2-40. VEGFR-3 was expressed in CD34-positive vessels in the tumor and in the normal adjacent tissue, whereas D2-40 staining was mainly observed in normal adjacent tissue. Black arrows indicate lymphatic vessels.
