# Supplementary material for: Targeting Tumor Angiogenesis with the Selective VEGFR-3 Inhibitor EVT801 in Combination with Cancer Immunotherapy
Source: Cancer Res Commun. 2022 Nov 29;2(11):1504–19. doi: 10.1158/2767-9764.CRC-22-0151 (PMC10035370; doi:10.1158/2767-9764.CRC-22-0151)
Supplement: Supplementary Figure S4 — shows the evaluation of EVT801’s pharmacokinetics and pharmacodynamics [file crc-22-0151-s05.docx]

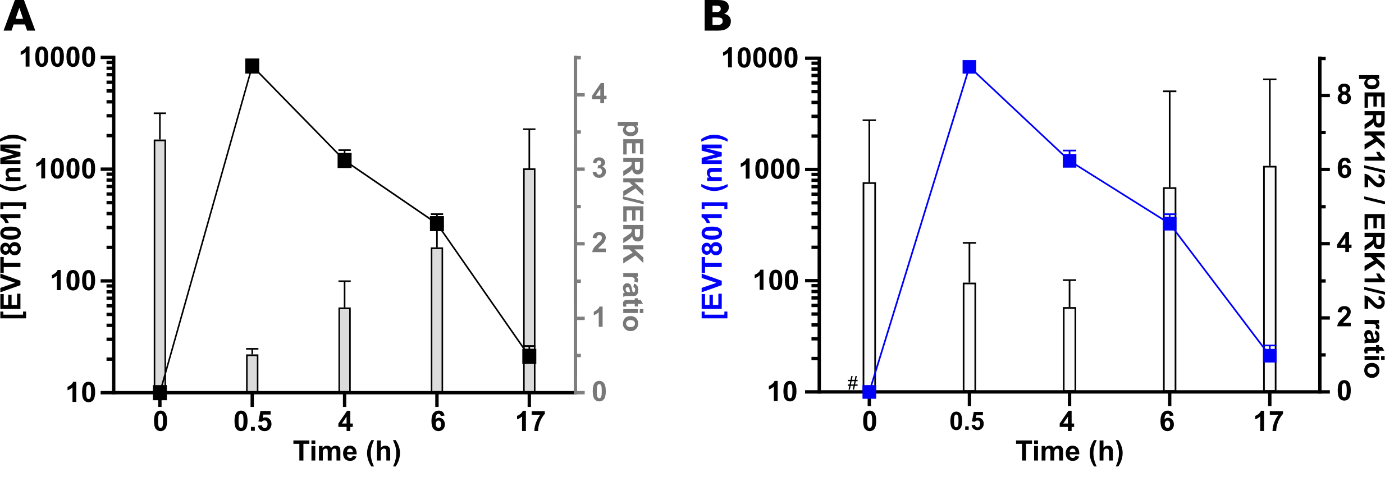


**Supplementary Figure 4.** Evaluation of pharmacokinetics and pharmacodynamics of EVT801. (A) Superposition of intratumor EVT801 concentration (black line) and ERK-phosphorylation (grey bars) over time indicates pharmacokinetics/pharmacodynamics correlation. (B) Superposition of intratumor EVT801 concentration (blue line) and ERK-phosphorylation (white bars) over time indicates pharmacokinetics/pharmacodynamics correlation.
