# Supplementary material for: Targeting Tumor Angiogenesis with the Selective VEGFR-3 Inhibitor EVT801 in Combination with Cancer Immunotherapy
Source: Cancer Res Commun. 2022 Nov 29;2(11):1504–19. doi: 10.1158/2767-9764.CRC-22-0151 (PMC10035370; doi:10.1158/2767-9764.CRC-22-0151)
Supplement: Supplementary Figure S5 — shows the immunohistochemical evaluation of hypoxia in NCI-H1703 subcutaneous tumor model [file crc-22-0151-s06.docx]

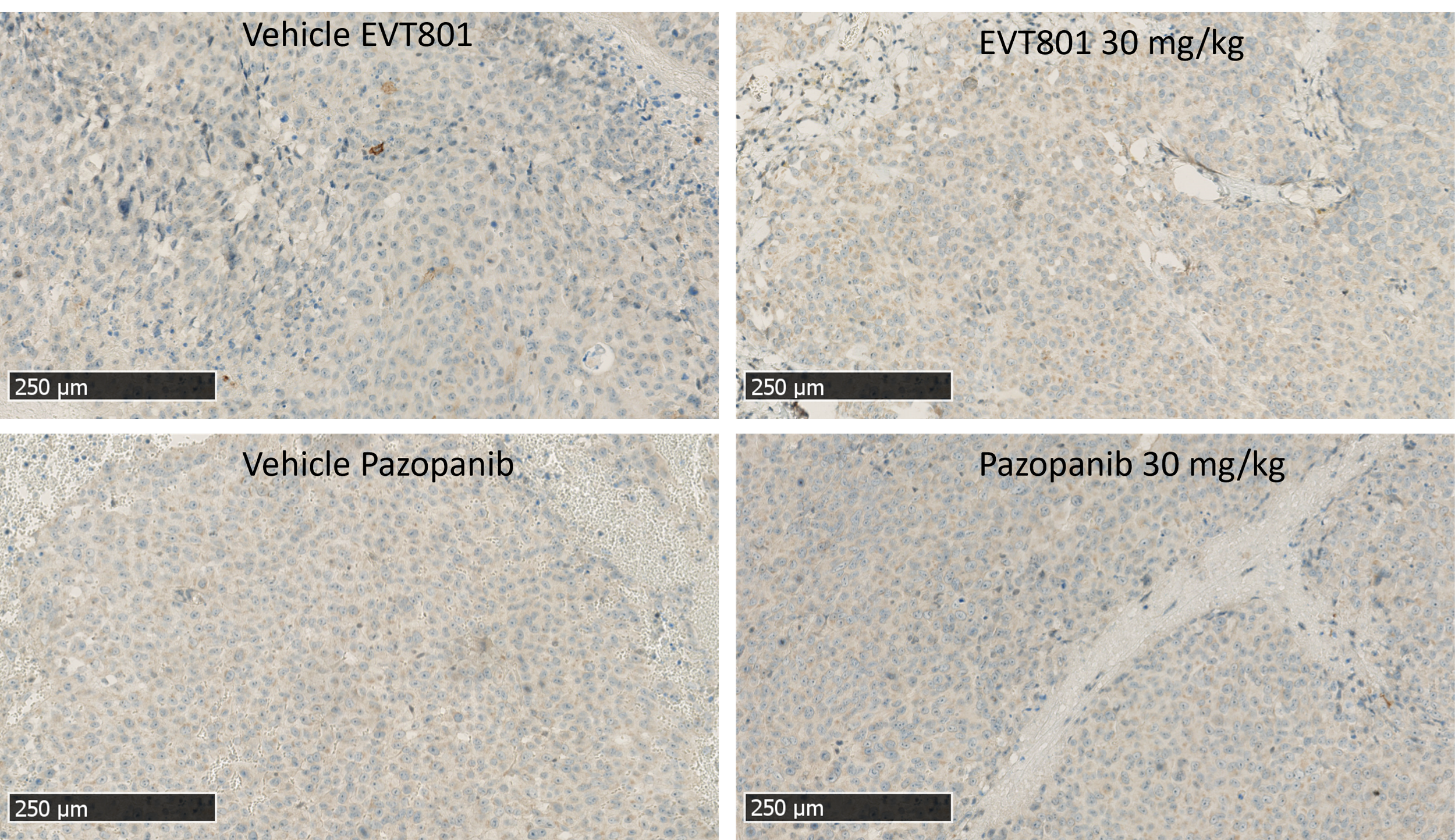


**Supplementary Figure 5.** Immunohistochemical evaluation of hypoxia in subcutaneous NCI-H1703 tumor xenografts after oral treatment with EVT801 and pazopanib.
