# Supplementary material for: Targeting Tumor Angiogenesis with the Selective VEGFR-3 Inhibitor EVT801 in Combination with Cancer Immunotherapy
Source: Cancer Res Commun. 2022 Nov 29;2(11):1504–19. doi: 10.1158/2767-9764.CRC-22-0151 (PMC10035370; doi:10.1158/2767-9764.CRC-22-0151)
Supplement: Supplementary Figure S6 — shows that EVT801 inhibits growth of VEGFR3-positive BNL hepatoma tumors, while decreasing vasculature and hypoxia [file crc-22-0151-s07.docx]

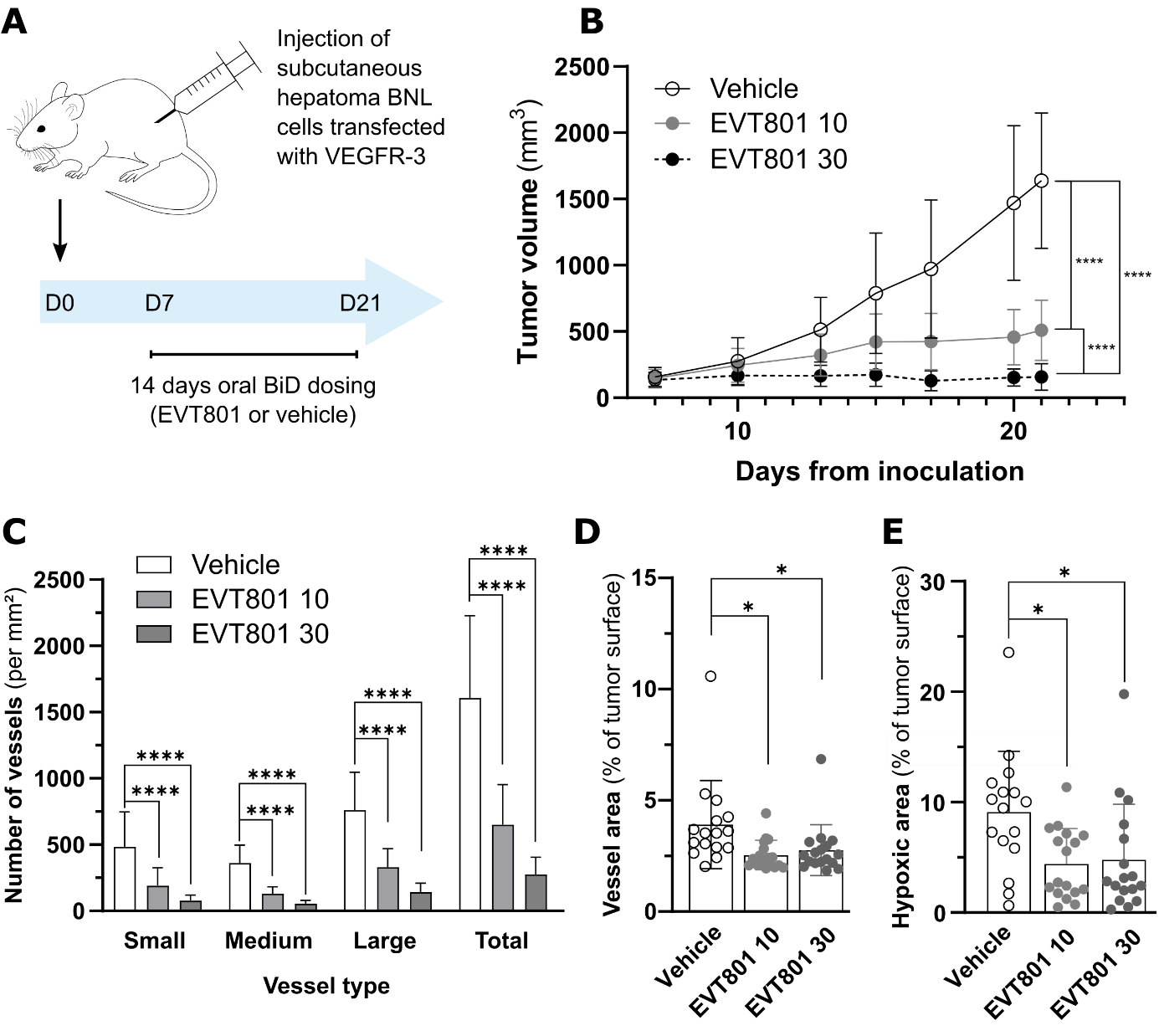


**Supplementary Figure 6.** EVT801 inhibits proliferation and growth of VEGFR3-positive BNL hepatoma cells. (A) Schematic view of the study protocol. BNL-R3 cells were injected subcutaneously in the flank of BALB/c mice and treated twice per day (BiD) from day 7 (D7) post-injection for 14 days with 10 mg/kg EVT801, 30 mg/kg EVT801 or vehicle. (B) Tumor volume progression over time in presence of 10 mg/kg EVT801, 30 mg/kg EVT801 or vehicle. (C) Small, medium, large and total blood vessel density in BNL-R3 tumors at end point after treatment with 10 mg/kg EVT801, 30 mg/kg EVT801 or vehicle. (D) Total vessel area, expressed as % of tumor surface, in BNL-R3 tumors at end point after treatment with 10 mg/kg EVT801, 30 mg/kg EVT801 or vehicle. (E) Surface of hypoxic zones in BNL-R3 tumors at end point after treatment with 10 mg/kg EVT801, 30 mg/kg EVT801 or vehicle.
