# Supplementary material for: Targeting Tumor Angiogenesis with the Selective VEGFR-3 Inhibitor EVT801 in Combination with Cancer Immunotherapy
Source: Cancer Res Commun. 2022 Nov 29;2(11):1504–19. doi: 10.1158/2767-9764.CRC-22-0151 (PMC10035370; doi:10.1158/2767-9764.CRC-22-0151)
Supplement: Supplementary Figure S7 — shows that EVT801 decreases immunosuppressive cell infiltration in VEGFR3-positive BNL hepatoma tumors [file crc-22-0151-s08.docx]

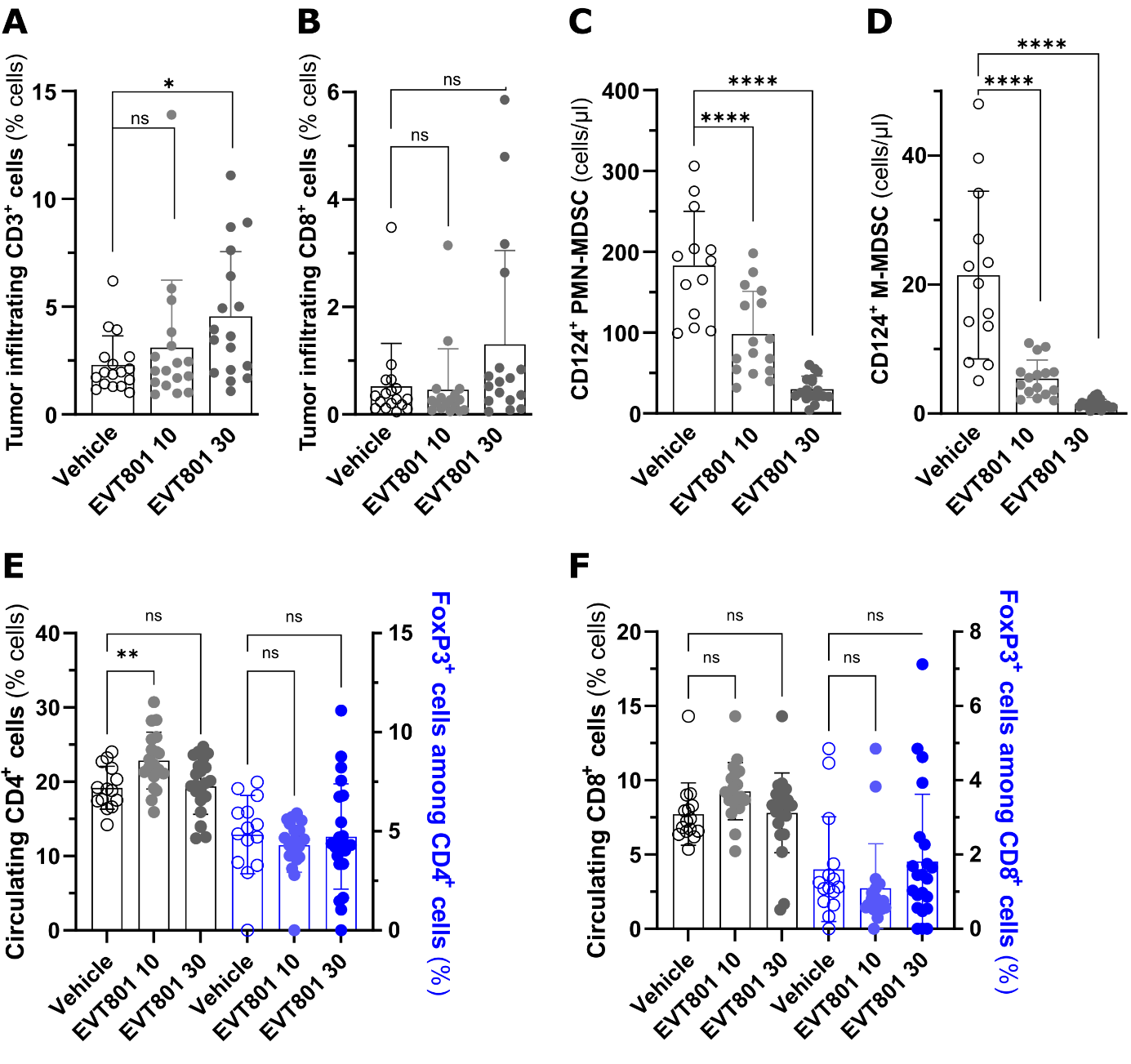


**Supplementary Figure 7.** EVT801 promotes immune cell infiltration into the tumor and reduces circulation of activated myeloid-derived immunosuppressor cells. (A) Relative amount of tumor infiltrating T cells (CD3^+^) in the BNL-R3 tumor mouse model at end point after treatment with 10 mg/kg EVT801, 30 mg/kg EVT801 or vehicle. (B) Relative amount of tumor infiltrating T cells (CD8^+^) in the BNL-R3 tumor mouse model at end point after treatment with 10 mg/kg EVT801, 30 mg/kg EVT801 or vehicle. (C) Concentration of CD124^+^ polymorphonuclear myeloid-derived suppressor cells (PMN-MDSCs) in the BNL-R3 tumor mouse model at end point after treatment with 10 mg/kg EVT801, 30 mg/kg EVT801 or vehicle. (D) Concentration of CD124^+^ polymorphonuclear myeloid-derived suppressor cells (M-MDSCs) in the BNL-R3 tumor mouse model at end point after treatment with 10 mg/kg EVT801, 30 mg/kg EVT801 or vehicle. (E) Relative amount of CD4^+^ T cells and the FoxP3^+^ T_reg_ cell subpopulation in the BNL-R3 tumor mouse model at end point after treatment with 10 mg/kg EVT801, 30 mg/kg EVT801 or vehicle. (F) Relative amount of CD8^+^ T cells and the FoxP3^+^ T_reg_ cell subpopulation in the BNL-R3 tumor mouse at end point after treatment with 10 mg/kg EVT801, 30 mg/kg EVT801 or vehicle.
