# Supplementary material for: Targeting Tumor Angiogenesis with the Selective VEGFR-3 Inhibitor EVT801 in Combination with Cancer Immunotherapy
Source: Cancer Res Commun. 2022 Nov 29;2(11):1504–19. doi: 10.1158/2767-9764.CRC-22-0151 (PMC10035370; doi:10.1158/2767-9764.CRC-22-0151)
Supplement: Supplementary Figure S8 — shows that EVT801 doesn’t modify blood pressure in rat while sorafenib increases it [file crc-22-0151-s09.docx]

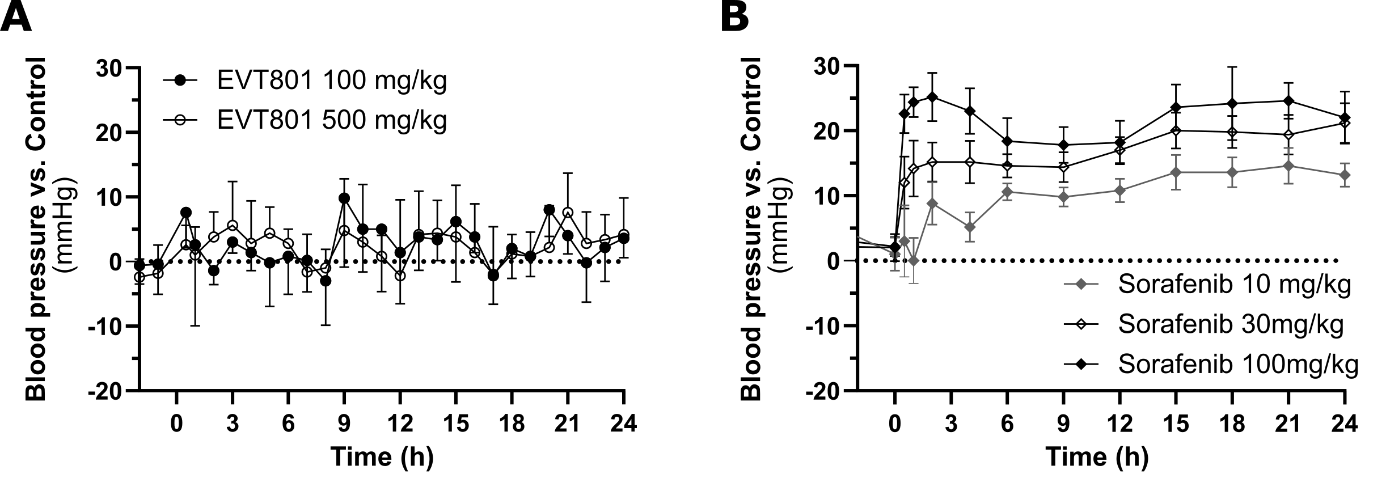


**Supplementary Figure 8.** EVT801 and sorafenib show opposing effects on blood pressure in rats. (A) Monitoring of the blood pressure over 24 h after single oral administration of 100 mg/kg EVT801 or 500 mg/kg of EVT801, compared to animals treated with vehicle. (B) Monitoring of the blood pressure over 24 h after single oral administration of 10 mg/kg sorafenib, 30 mg/kg sorafenib or 100 mg/kg of sorafenib, compared to animals treated with vehicle.
