# Supplementary material for: Targeting Tumor Angiogenesis with the Selective VEGFR-3 Inhibitor EVT801 in Combination with Cancer Immunotherapy
Source: Cancer Res Commun. 2022 Nov 29;2(11):1504–19. doi: 10.1158/2767-9764.CRC-22-0151 (PMC10035370; doi:10.1158/2767-9764.CRC-22-0151)
Supplement: Supplementary Figure S9 — provides an overview of the mechanisms by which EVT801 exerts its anti-tumor effects [file crc-22-0151-s10.docx]

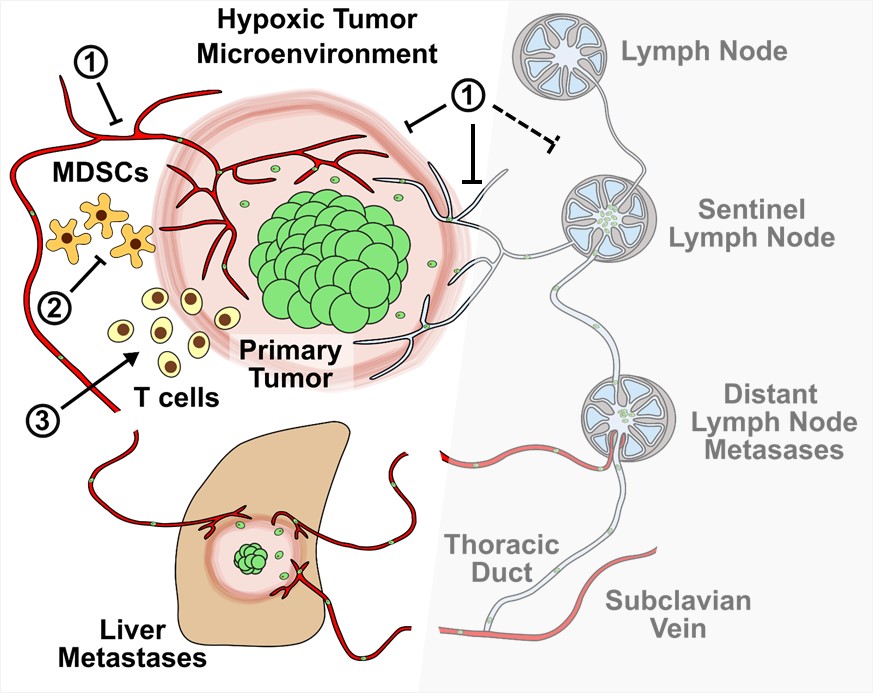


**Supplementary Figure 9.** Overview of the anti-tumor effects of EVT801. The figure summarizes the three modes-of-action of EVT801 against tumors, supporting evaluation of EVT801 in patients, either as stand-alone treatment or in combination with immune checkpoint inhibitors:

(1) Inhibition of tumor escape and metastasis via stabilization of tumor vasculature, inhibition of lymphangiogenesis and reduction of hypoxia in the tumor microenvironment.

(2) Strengthening of anti-tumor immunity via reduction of MDSCs.

(3) Enhancement of anti-tumor immunity by increasing infiltration of T cells.

Precise mechanisms leading to reduction of distant metastasis (greyed out) are proposed but yet to be confirmed.
