# Supplementary material for: Targeting Tumor Angiogenesis with the Selective VEGFR-3 Inhibitor EVT801 in Combination with Cancer Immunotherapy
Source: Cancer Res Commun. 2022 Nov 29;2(11):1504–19. doi: 10.1158/2767-9764.CRC-22-0151 (PMC10035370; doi:10.1158/2767-9764.CRC-22-0151)
Supplement: Supplementary Table TS1 — Supplementary Table S1 shows EVT801 potency and selectivity profile [file crc-22-0151-s11.docx]

**Table S2. Evaluation of EVT801 metabolite**

| Evaluation of SAR401849 | Species/Target | Assay | Result |
| --- | --- | --- | --- |
|  |  |  |  |
| VEGFR-1/-2/-3 autophosphorylation | Human | *In vitro* assay  Cellular assay | 823 nM / 244 nM / 19.7 nM  2270 nM / 210 nM / 32.4 nM |
| VEGFR-3 autophosphorylation | Mouse | Cellular assay | 63 nM |
|  | Rat |  | 91.4 nM |
|  | Dog |  | 44.7 nM |
|  | Monkey |  | 34.2 nM |
| Selectivity | 48 or 60 kinase panel | Internal kinase panel ^a^ | VEGFR2 (90 %)  AuroraB (50 %)  VEGFR1 (61 %)  LRRK2 (54 %) |
|  | 107 targets | CEREP | A3  A2a  A1  PDE3A ^b^ |
| Ion channels | 7 targets | ^c^ | No effect |
| ^a^ inhibition at 1 µM; ^b^ (> 80 % inhibition at 10 µM); ^c^ concentration of 1 µM | | | |

**Table S3. Half-life and tissue/tumor distribution in rodents**

| Species / strain | Dose regimen | T_1/2_ in plasma (h)^a^ | Tissue to plasma (AUC) |
| --- | --- | --- | --- |
|  |  |  |  |
| Balb/c mice | 30 mg/kg  100 mg/kg | 2.7  1.1 | N.D  N.D |
| Balb/c with 4T1 tumor  SCID mice with tumor  Sprague-Dawley rats | 100 mg/kg for 2 weeks | 3.2  0.9 (EVT801)  2.6 (SAR849)  1.6 (EVT801)  1.8 (SAR849)  2.4 | 0.88 |
|  | 10 mg/kg for 2 weeks  30 mg/kg for 2 weeks |  | 2.6  1.4  2.2  1.3 |
|  | 10 mg/kg |  | Kidney: 1.0  Liver: 1.5  Brain: 0.14  Heart: 0.46 |
| ^a^ Only EVT801 was quantified if not specified otherwise. AUC, area under curve; N.D., not determined | | | |
